# The Efficacy and Tolerability of Selective Serotonin Reuptake Inhibitors for Motor Recovery in Non-depressed Patients After Acute Stroke: A Meta-Analysis

**DOI:** 10.3389/fneur.2021.749322

**Published:** 2021-10-20

**Authors:** Ning Su, Changming Wen, Shiqian Guo, Yang Yu, Chenglin Wang

**Affiliations:** Department of Neurology, Nanyang Central Hospital, Nanyang, China

**Keywords:** efficacy, tolerability, selective serotonin reuptake inhibitors, stroke, meta-analysis

## Abstract

**Objective:** To explore the efficacy and tolerability of selective serotonin reuptake inhibitors (SSRIs) for motor recovery in non-depressed patients after acute stroke.

**Methods:** According to the predefined retrieval strategy, multiple electronic databases were searched for randomized controlled trials (RCTs) that met the inclusion criteria. The primary efficacy outcome was measured by Fugl-Meyer Motor Scale (FMMS) score and the indicators of tolerability included withdrawal rate and the incidence of adverse events (AEs).

**Results:** 10RCTs were included, the pooled analyses showed patients who received fluoxetine (endpoint: MD = 21.17, 95% CI 14.13–28.21, *P* < 0.00001; mean change: MD = 16.27, 95% CI 10.05–22.50, *P* < 0.00001) and citalopram (endpoint: MD = 22.93, 95% CI 11.13–34.73, *P* = 0.0001; mean change: MD = 24.06, 95% CI 10.47–37.65, *P* = 0.0005) experienced greater improvement in FMMS score. There was no evident difference in total withdrawal rate (fluoxetine: OR = 1.11, 95% CI 0.90–1.27, *P* = 1.38; citalopram: OR = 0.94, 95% CI 0.69–1.28, *P* = 0.71; escitalopram: OR = 0.87, 95% CI 0.58–1.28, *P* = 0.47) between two groups. Besides, the incidence of hyponatremia (OR = 2.01, 95% CI 1.16–3.50, *P* = 0.01), seizure (OR = 1.46, 95% CI 1.03–2.08, *P* = 0.04) and fracture (OR = 2.34, 95% CI 1.61–3.40, *P* < 0.00001) in the fluoxetine group was higher than in the placebo group.

**Conclusions:** Fluoxetine and citalopram can promote motor recovery in non-depressed patients with acute stroke, but it is necessary to pay attention to the possible AEs of fluoxetine, such as hyponatremia, seizure and fracture.

**Systematic Review Registration:** PROSPERO, identifier [CRD42021227452].

## Introduction

Stroke is the second leading cause of death in the world and a leading cause of long-term functional disability (1). Stroke affects 13.7 million people each year, and approximately half of all survivors are left with a disability (2, 3). In recent years, although great progress has been made in the treatment of acute stroke, post-stroke disability is still an urgent problem to be solved.

Many clinical studies have suggested that selective serotonin reuptake inhibitors (SSRIs) might improve functional outcomes after stroke, even in non-depressed patients, through a range of mechanisms, which include enhancing neuroplasticity and promoting neurogenesis (4–6). The previous systematic review (7) has shown that SSRIs seem to reduce disability, dependence, and neurological impairment after stroke. Based on the results of this systematic review (7), three large randomized controlled trials (RCTs) (5, 6, 8) have been designed. However, the FOCUS trial does not demonstrate any benefit on the functional outcome of fluoxetine compared with placebo at 6 months. A subsequent systematic review (9) was conducted based on the previous work, which only included the trials at low risk of bias and the FOCUS, and the results indicated that SSRIs did not improve recovery from stroke.

Given this situation, and two other large studies that have been published, we intend to conduct a meta-analysis and systematically review the relevant literature to evaluate the efficacy and tolerability of SSRIs for motor recovery in non-depressed patients after acute stroke to provide evidence for clinical treatment.

## Methods

The following work was conducted according to the preferred reporting items for systematic reviews and meta-analyses (PRISMA) statement (10). The PROSPERO registration number is CRD42021227452.

### Criteria for Considering Studies for This Review

The inclusion criteria were as follows: (1) participants: patients aged 18 years or older with acute post-stroke hemiparesis or hemiplegia and Fugl-Meyer motor scale (FMMS) scores ≤ 55 or the mRS score ≥1; (2) intervention: one of the SSRIs was used as monotherapy, and there was no limit on the dosage and dosage form of them; (3) comparison: placebo; (4) outcome measures: indicators that could reflect efficacy or tolerability. As long as one of these indicators was present in the study, it was considered appropriate; (5) study types: RCTs.

Studies were excluded if they included patients with: (1) depression; (2) severe physical disease; (3) poor response or contraindications to SSRIs previously; (4) severe post-stroke disability or premorbid disabilities including aphasia, cognitive disorders, and motor disorders. Moreover, duplicate publications and studies without available data would also be ruled out.

### Search Methods for Identification of Studies

PubMed, Web of Science, Embase, Cochrane Central Register of Controlled Trials, ScienceDirect and Scopus were searched from their inception to November 6, 2020. Only articles published in English would be included. The search terms were: (stroke OR “cerebrovascular accident” OR “cerebral infarction” OR “brain infarction” OR “cerebral hemorrhage” OR “intracranial hemorrhages”) AND (“serotonin uptake inhibitors” OR citalopram OR escitalopram OR fluoxetine OR paroxetine OR sertraline OR fluvoxamine). We used a combination of Medical Subject Headings (MESH) words and free words for retrieval, and made corresponding adjustments according to the characteristics of each database. We also searched Clinical Trials.gov to seek the unpublished studies. Details of the search strategy were provided in Supplementary File 1. In order not to omit relevant studies, we would also manually retrieve all references of the included literature, as well as those in the systematic reviews and meta-analyses related to the research topic. Finally, we searched again on June 22, 2021, to avoid missing new literature published after the end of our search.

### Data Collection and Analysis

#### Selection of Studies

After removing the duplicate literature, two researchers removed the studies that did not meet the inclusion criteria by reading the titles and abstracts of them, and then read the full text of the remaining studies in detail to decide whether they could be included in our study. If there was any disagreement, they should consult with the third researcher to make a decision finally.

#### Data Extraction and Management

These data were summarized in an Excel spreadsheet: the first authors, publication year, study design, numbers/gender/age of the participants, stroke type, intervention and control measures, duration of treatment, indicators of efficacy and tolerability. Two researchers performed this work independently and a third people helped them solve any controversy that may arise.

#### Assessment of Risk of Bias in Included Studies

We evaluated the quality of the included RCTs based on the Cochrane Collaboration's tool (11), which mainly involved the following six items: selection bias (random sequence generation and allocation concealment), performance bias (blinding of participants and personnel), detection bias (blinding of outcome assessment), attrition bias (incomplete outcome data), reporting bias (selective reporting), and other biases. The risk of bias for each project is expressed as “low risk,” “high risk” or “unclear risk.” Among them, “high risk of bias” means that it may alter the outcome seriously, “low risk of bias,” if present, is unlikely to alter the results seriously, “unclear risk of bias” means it would raise some doubt about the results (11). Two authors evaluated the risk of bias on their own, if there was a disagreement, they could discuss it with a third person.

#### Measures of Treatment Effect

FMMS is a well-designed, feasible, and efficient clinical examination method that is widely used for assessment of motor recovery after stroke and has excellent intra-rater and inter-rater reliability and validity (12). Thus, the primary efficacy outcome was measured by FMMS score as well as the secondary efficacy outcomes included Barthel Index (BI) score, National Institutes of Health Stroke Scale (NIHSS) score, modified Rankin Scale (mRS) score, and the proportion of patients with a mRS score of 0–2. Besides, the indicators of tolerability included withdrawal rate and the incidence of adverse events (AEs).

#### Unit of Analysis Issues

Only randomized, placebo-controlled, parallel-group trials would be included. When trials had diverse treatment arms with different doses or different types of antidepressants, we would compare them with placebo, respectively.

#### Dealing With Missing Data

For the missing data, we would first try to contact the original authors to get the corresponding data, if not, for dichotomous variables, if there was a lack of one of the number of events or the total number of events in the literature, and the incidence of events was provided, the missing data could be improved by simple conversion. For continuous variables, if the mean and standard deviation (SD) were not provided but only the median and Interquartile Range (IQR) were mentioned in the literature, we would estimate according to the methods mentioned in the studies (13, 14).

#### Assessment of Heterogeneity

The *P*-value of the chi-square test and I-square (*I*^2^) were used to assess heterogeneity. Low, moderate, and high heterogeneity were defined as *I*^2^ values of 25, 50, and 75%, respectively (15). As for this study, heterogeneity was acceptable as long as *I*^2^ is not >50%. *P* = 0.10 was set as the threshold for statistical significance (16).

#### Assessment of Reporting Biases

Our meta-analysis mainly involved publication bias and selective outcome reporting bias. If we had at least 10 studies in the meta-analysis, we would use a funnel plot to qualitatively analyze publication bias, besides, the Begg's and Egger's test would be exploited for potential publication bias assessment quantificationally, and *P*-value < 0.05 would be interpreted as statistically significant (17). To examine the presence of selective outcome reporting bias, we compared the specified outcomes in the trial registry, if they were available, with those reported in the published studies. If not, we would scrutinize the aims and methods of the studies and comparing these with outcomes reported. We determined that if published reports included all prespecified outcomes, then those studies had a low risk of bias.

#### Data Synthesis

The RevMan 5.3 software from the Cochrane Collaboration was applied for the data synthesis. We pooled mean difference (MD) for continuous data using Inverse Variance methods and odds ratios (OR) for dichotomous data using Mantel-Haenszel methods, reporting pooled results along with their associated 95% confidence intervals (CI). If the *P* < 0.10 or *I*^2^ ≥ 50%, substantial heterogeneity between studies was indicated, and the random-effects model was used; otherwise, we used the fixed-effect model (18). A *P*-value < 0.05 in the overall effect *Z* test was considered statistically significant (19).

#### Subgroup Analysis and Investigation of Heterogeneity

Subgroup analysis was often used to deal with heterogeneity. When significant heterogeneity (*I*^2^ > 50%) was found in this study, in addition to using the random-effects model, if the number of studies was sufficient, we could conduct subgroup analysis to explore the source of heterogeneity based on the types of antidepressants.

#### Sensitivity Analysis

In order to evaluate the robustness of the final results and explore the contribution of each included trial, we would conduct the sensitivity analysis. Stata 15.1 software was used to recalculate the data after removing the study item by item and observe whether the final statistical results changed or not. Finally, the sensitivity analysis chart was drawn.

## Results

### Description of Studies

#### Results of the Search

The literature search identified a total of 1,344 records from electronic databases and the reference lists of retrieved studies, relevant systematic reviews, and meta-analyses.

**Figure 1:**
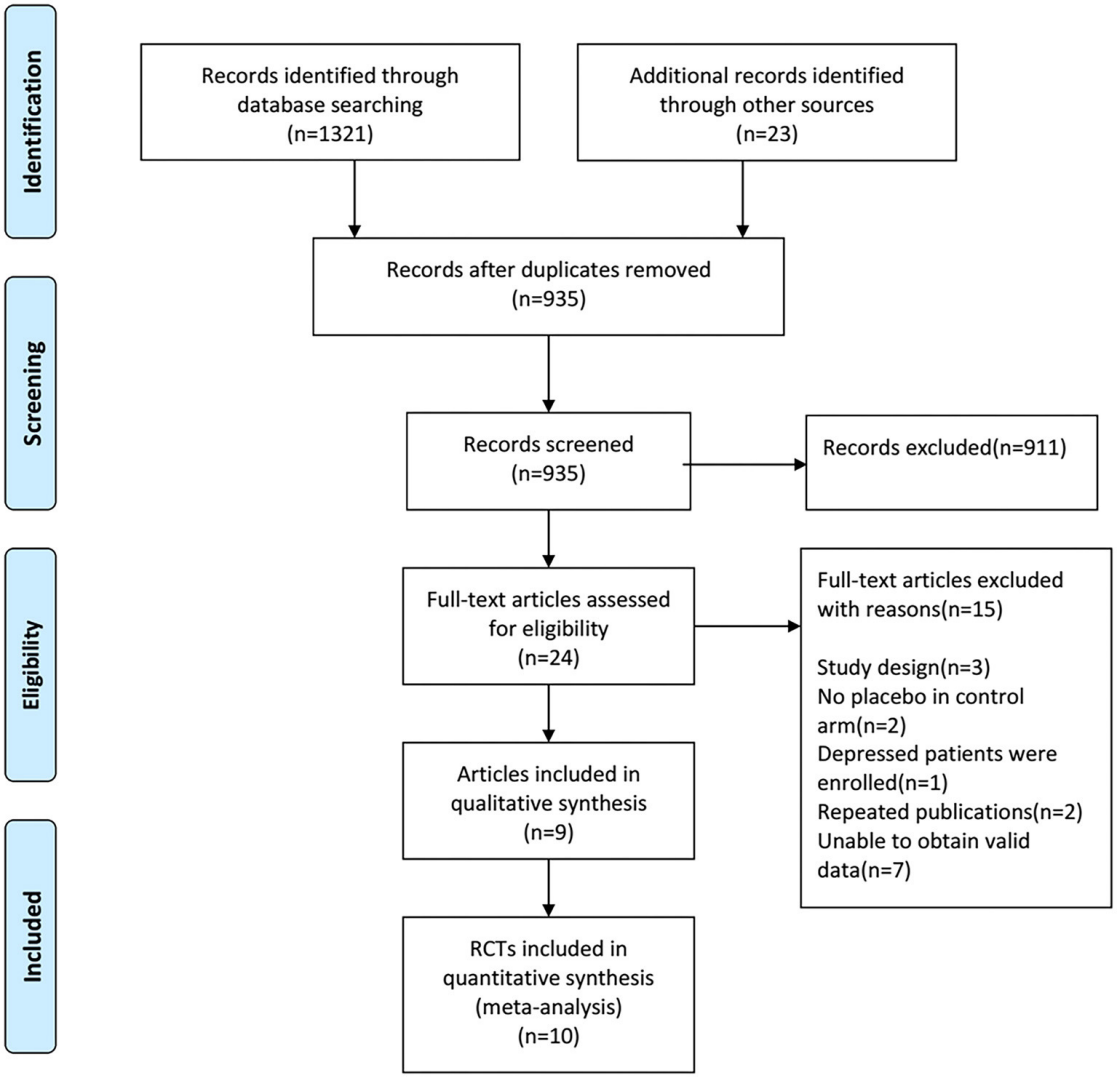
PRISMA flow diagram.

We used EndNote X9 software to remove duplicate records and carry out manual re-inspection, the 409 duplicate records were removed. We scanned the titles and abstracts of the remaining 935 documents, 911 records that did not meet the inclusion criteria were removed. Then the full-text of 24 articles were carefully evaluated for their eligibility, 15 articles were excluded and 9 studies were included for qualitative synthesis, in addition, 10 RCTs were included in our meta-analysis finally. See Figure 1 for PRISMA flow diagram.

#### Included Studies

The main characteristics of the included studies were shown in Table 1. All included studies were randomized, double-blind, placebo-controlled trials. These studies were published between 2011 and 2020. The sample size ranged from 32 to 3,127. There were 3,722 participants in the experimental group and 3,689 participants in the control group. Mean age ranged from 58.7 to 71.5 years. The experimental groups of the 10 RCTs were, respectively, 20 mg fluoxetine in 6 RCTs, 20 mg citalopram in 3 RCTs, and 10 mg escitalopram in 1RCT. The course of treatment ranged from 12 weeks to 6 months.

**Table 1:** The characteristics of included RCTs in the meta-analysis.

**References**	**Case (I/C)**	**Gender (M/F)**	**Age (I/C) (years)**	**Stroke type**	**Intervention/ control**	**Dose (mg/d)**	**Durance**	**Outcome**	**Multicenter**	**Study design**
Asadollahi et al. (20)	60 (30/30)	33/27	60.2 ± 8.52/61.7 ± 9.6	Ischemic stroke	Fluoxetine/placebo	20	90d	(1), (6), (7)	N	RCT
Asadollahi et al. (20)	60 (30/30)	35/25	58.7 ± 8.56/61.7 ± 9.6	Ischemic stroke	Citalopram/placebo	20	90d	(1), (6), (7)	N	RCT
Chollet et al. (4)	118 (59/59)	72/46	66.4 ± 11.7/62.9 ± 13.4	Ischemic stroke	Fluoxetine/placebo	20	90d	(1), (3), (5), (6), (7)	Y	RCT
FOCUS Trial Collaboration (5)	3,127 (1,564/1,563)	1,922/1,205	71.2 ± 12.4/71.5 ± 12.1	Intracerebral hemorrhage or ischaemic stroke	Fluoxetine/placebo	20	6 months	(5), (6), (7)	Y	RCT
Kraglund et al. (21)	642 (319/323)	421/221	68 ± 13/68 ± 13	Ischemic stroke	Citalopram/placebo	20	6 months	(4), (5), (6), (7)	Y	RCT
Affinity Trial Collaboration (6)	1,280 (642/638)	804/476	63.5 ± 12.5/64.6 ± 12.2	Intracerebral hemorrhage or ischaemic stroke	Fluoxetine/placebo	20	6 months	(5), (6), (7)	Y	RCT
Kim et al. (22)	478 (241/237)	–	–	Intracerebral hemorrhage or ischaemic stroke	Escitalopram/placebo	10	12 weeks	(2), (3), (4), (6), (7)	Y	RCT
Effects Trial Collaboration (8)	1,500 (750/750)	925/575	70.6 ± 11.3/71.0 ± 10.5	Intracerebral hemorrhage or ischaemic stroke	Fluoxetine/placebo	20	6 months	(3), (5), (6), (7)	Y	RCT
Marquez-Romero et al. (23)	32 (15/17)	15/15	–	Intracerebral hemorrhage	Fluoxetine/placebo	20	90d	(1), (2), (3), (5), (6), (7)	Y	RCT
Savadi Oskouie et al. (24)	144 (72/72)	–	–	Ischemic stroke	Citalopram/placebo	20	90d	(5), (6), (7)	N	RCT

*RCTs, randomized controlled trials; I/C, intervention/control; M, male; F, female; Y, yes; N, no; (1) Fugl-Meyer Motor Scale (FMMS) score; (2) Barthel Index (BI) score; (3) National Institutes of Health Stroke Scale (NIHSS) score; (4) modified Rankin Scale (mRS) score; (5) the proportion of patients with a mRS score of 0–2; (6) total withdrawal rate; (7) the incidence of adverse events (AEs)*.

#### Excluded Studies

We excluded 15 articles for the following reasons: (1) were non-randomized controlled trials (*n* = 3), (2) no placebo in control arm (*n* = 2), (3) depressed patients were enrolled (*n* = 1), (4) repeated publications (*n* = 2), (5) unable to obtain valid data (*n* = 7).

### Risk of Bias in Included Studies

**Figure 2:**
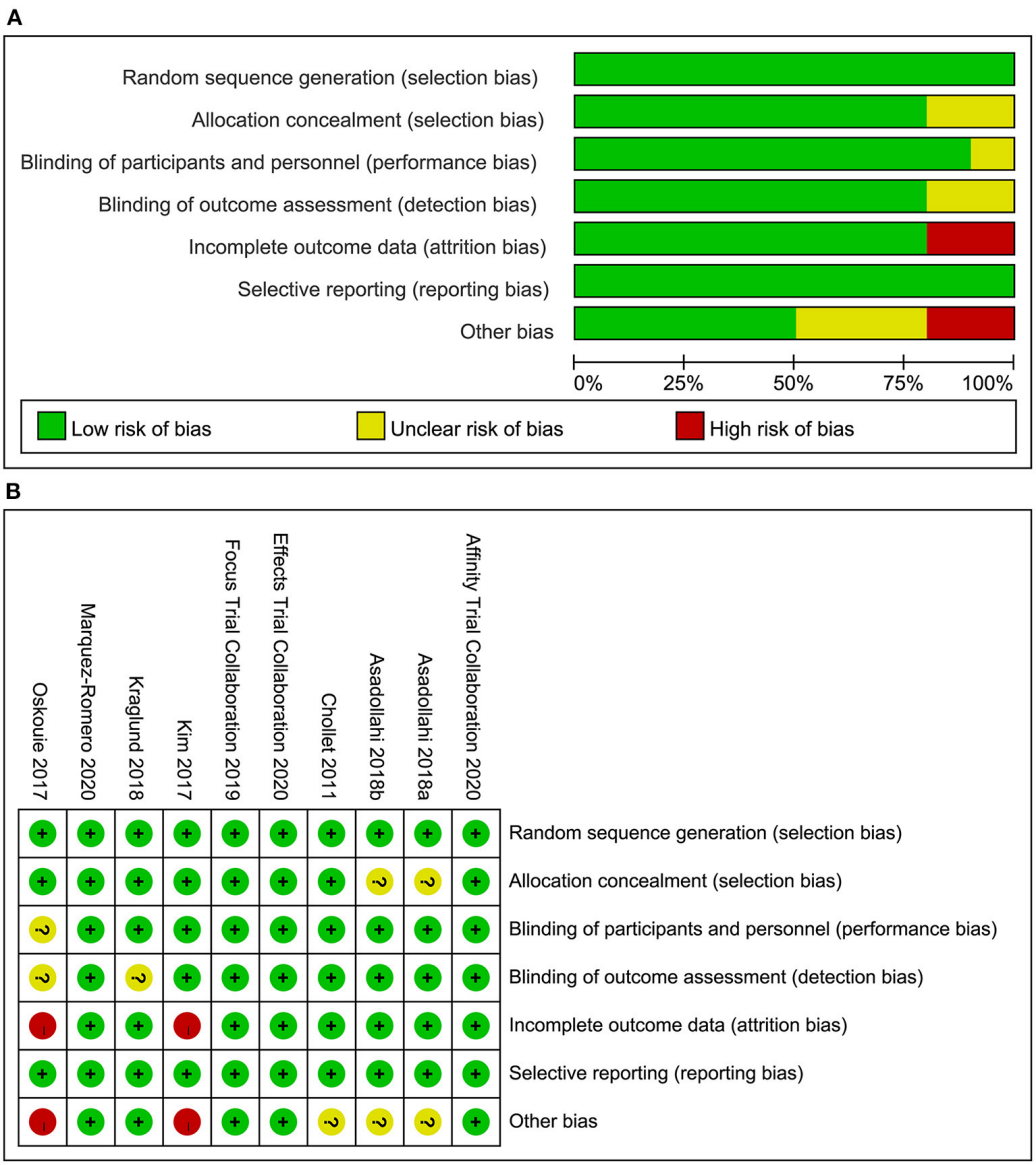
**(A)** Risk of bias graph: review authors' judgements about each risk of bias item presented as percentages across all included studies, **(B)** Risk of bias summary: review authors' judgements about each risk of bias item for each included study.

Risk of bias assessment of the included studies was presented in Figure 2 (A: risk of bias graph; B: risk of bias summary).

### Effects of Interventions

#### Primary Efficacy Outcomes

##### FMMS-Endpoint Score

**Figure 3:**
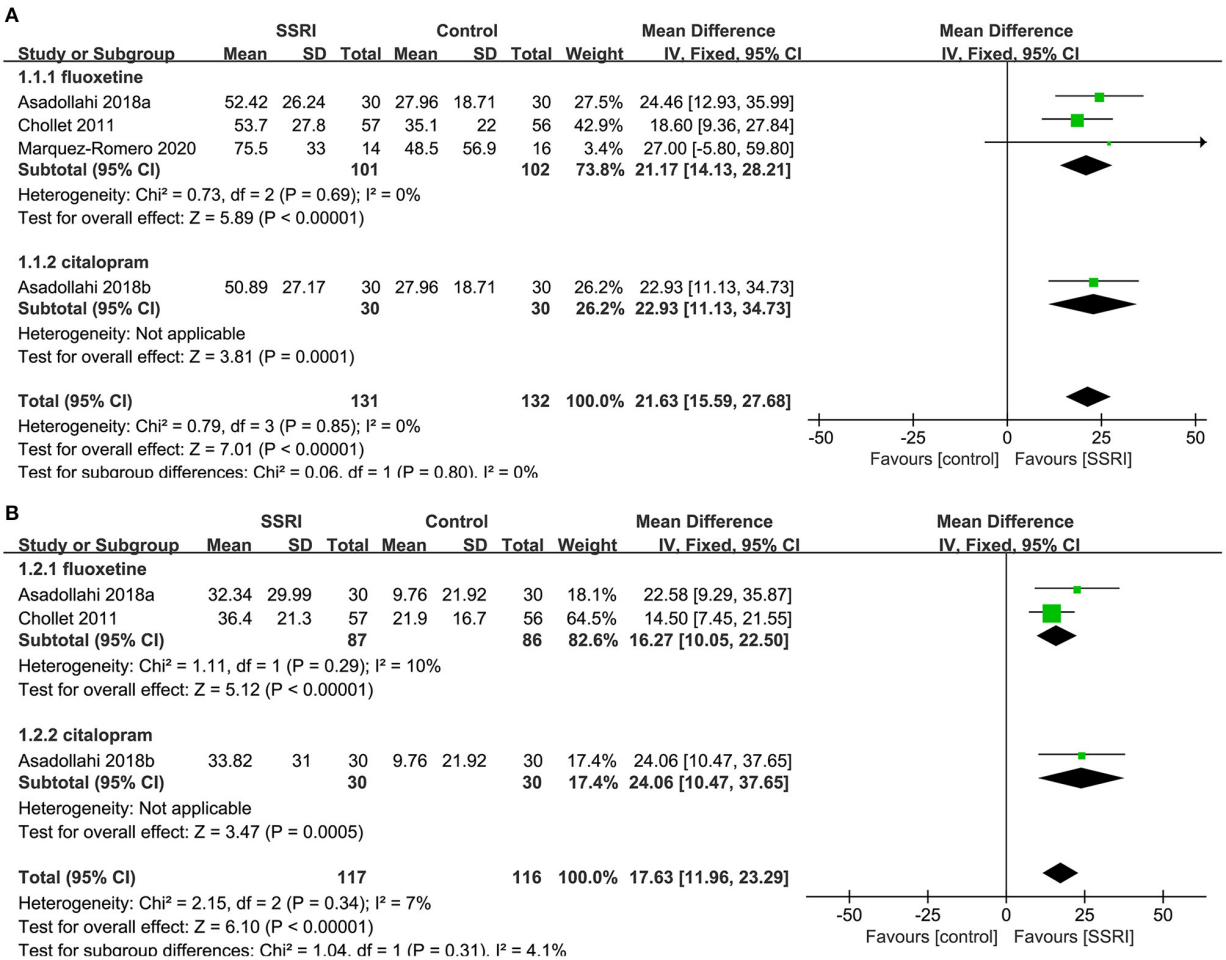
Forest plot of primary efficacy outcomes. **(A)** FMMS-endpoint score, **(B)** FMMS-mean change. FMMS, Fugl-Meyer Motor Scale.

We found evidence showing a benefit for fluoxetine (4, 20, 23) compared to placebo (3RCTs, MD = 21.17, 95% CI 14.13–28.21, *P* < 0.00001). No heterogeneity was detected (*P* = 0.69, *I*^2^ = 0%).One study on citalopram (20) showed a benefit for the drug compared to placebo (MD = 22.93, 95% CI 11.13–34.73, *P* = 0.0001) (Figure 3A).

##### FMMS-Mean Change

We found evidence showing a benefit for fluoxetine (4, 20) compared to placebo (2RCTs, MD = 16.27, 95% CI 10.05–22.50, *P* < 0.00001). No heterogeneity was detected (*P* = 0.29, *I*^2^ = 10%).One study on citalopram (20) showed a benefit for the drug compared to placebo (MD = 24.06, 95% CI 10.47–37.65, *P* = 0.0005) (Figure 3B).

#### Secondary Efficacy Outcomes

##### The Proportion of the Number of People With mRS Score 0–2

**Figure 4:**
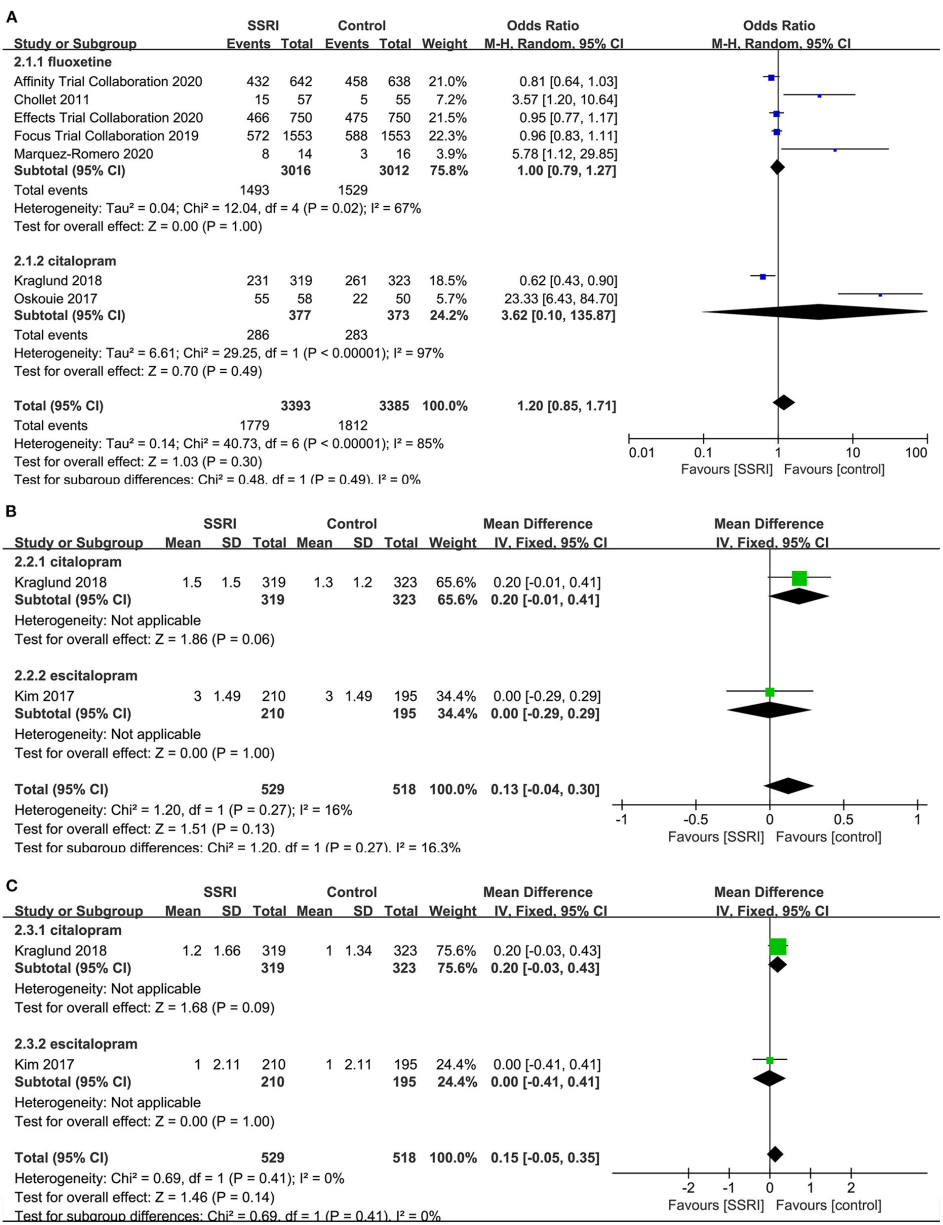
Forest plot of secondary efficacy outcomes. **(A)** The proportion of the number of people with mRS score 0–2, **(B)** mRS-endpoint score, **(C)** mRS-mean change. mRS, modified Rankin Scale.

There was no significant difference in the proportion of patients with a mRS score of 0–2 at the end of treatment between the fluoxetine (4–6, 8, 21] (5RCTs, OR = 1.00, 95% CI 0.79–1.27, *P* = 1.00) and citalopram groups (21, 24) (2RCTs, OR = 3.62, 95% CI 0.10–135.87, *P* = 0.49) compared to the placebo group. The heterogeneity of the two groups was significant (fluoxetine: *P* = 0.02, *I*^2^ = 67%; citalopram: *P* < 0.00001, *I*^2^ = 97%), so the random-effects model was applied (Figure 4A).

##### mRS-Endpoint Score

A total of two studies reported relevant data, namely citalopram (21) (MD = 0.20, 95% CI −0.01 to 0.41, *P* = 0.06) and escitalopram (22) (MD = 0.00, 95% CI −0.29 to 0.29, *P* = 1.00) in the treatment group, and the results showed no statistical difference compared with the control group (Figure 4B).

##### mRS-Mean Change

There was no significant difference in the mean change of mRS between citalopram (21) (MD = 0.20, 95% CI −0.03 to 0.43, *P* = 0.09) and escitalopram (22) (MD = 0.00, 95% CI −0.41 to 0.41, *P* = 1.00) groups at the end of treatment compared with the control group (Figure 4C).

##### NIHSS-Endpoint Score

**Figure 5:**
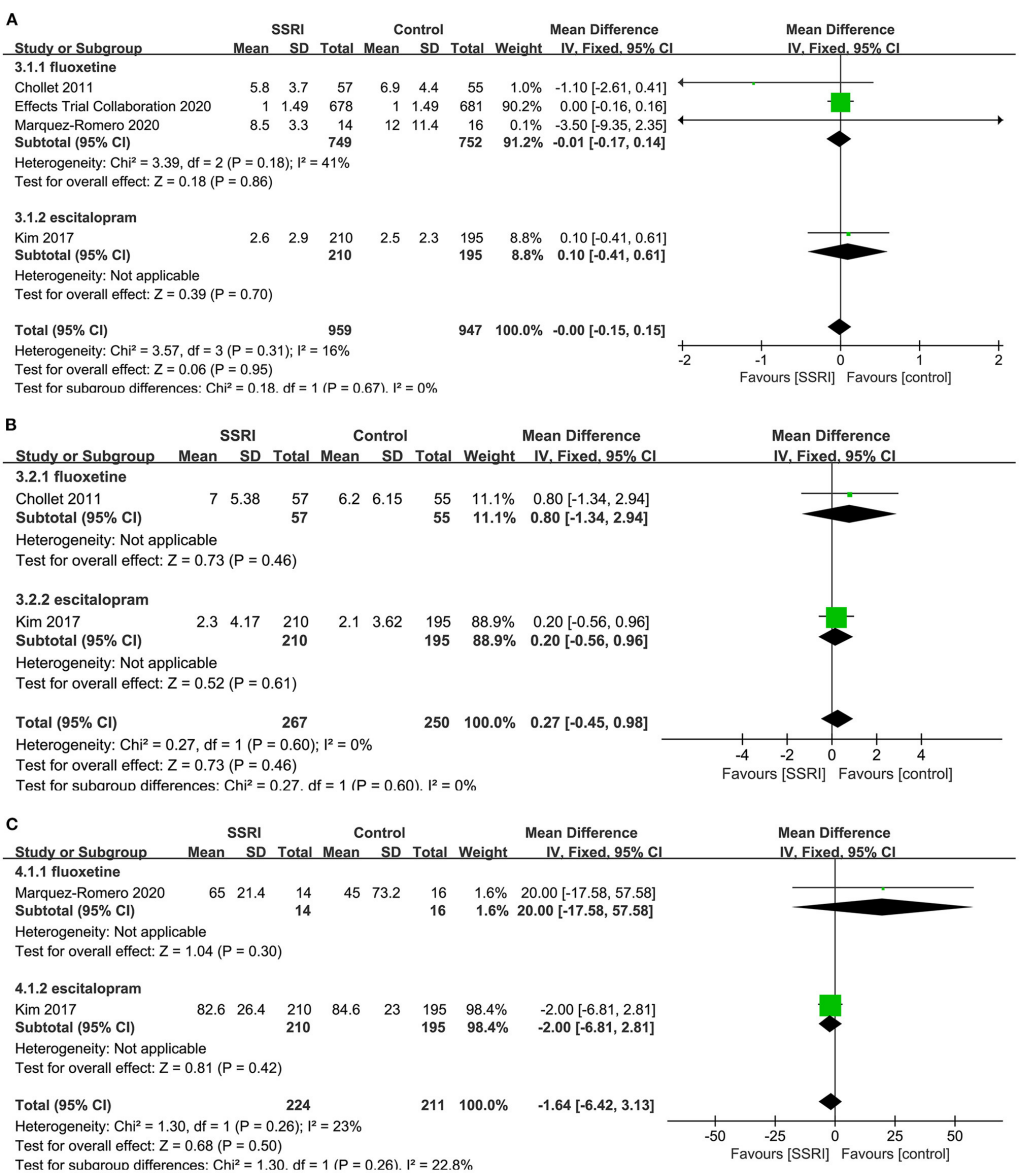
Forest plot of secondary efficacy outcomes. **(A)** NIHSS-endpoint score, **(B)** NIHSS-mean change, **(C)** BI-endpoint score. NIHSS, National Institutes of Health Stroke Scale; BI, Barthel Index.

There was no statistically significant difference in NIHSS score at the end of treatment between fluoxetine (4, 8, 23) (3RCTs, MD = −0.01, 95% CI −0.17 to 0.14, *P* = 0.86) and placebo. No significant heterogeneity was found (*P* = 0.18, *I*^2^ = 41%). One study found no significant difference between escitalopram (22) (MD = 0.10, 95% CI −0.41 to 0.61, *P* = 0.70) and placebo (Figure 5A).

##### NIHSS-Mean Change

There was no evidence that fluoxetine (4) (MD = 0.80, 95% CI −1.34 to 2.94, *P* = 0.46) and escitalopram (22) (MD = 0.20, 95% CI −0.56 to 0.96, *P* = 0.61) changed NIHSS score significantly at the end of treatment compared to the control group (Figure 5B).

##### BI-Endpoint Score

A total of two studies reported relevant data, namely fluoxetine (23) (MD = 20.00, 95% CI −17.58 to 57.58, *P* = 0.30) and escitalopram (22) (MD = −2.00, 95% CI −6.81 to 2.81, *P* = 0.42) in the treatment group, and the results showed no statistical difference compared with the control group (Figure 5C).

#### The Tolerability Outcomes

##### Withdrawal Rate

**Figure 6:**
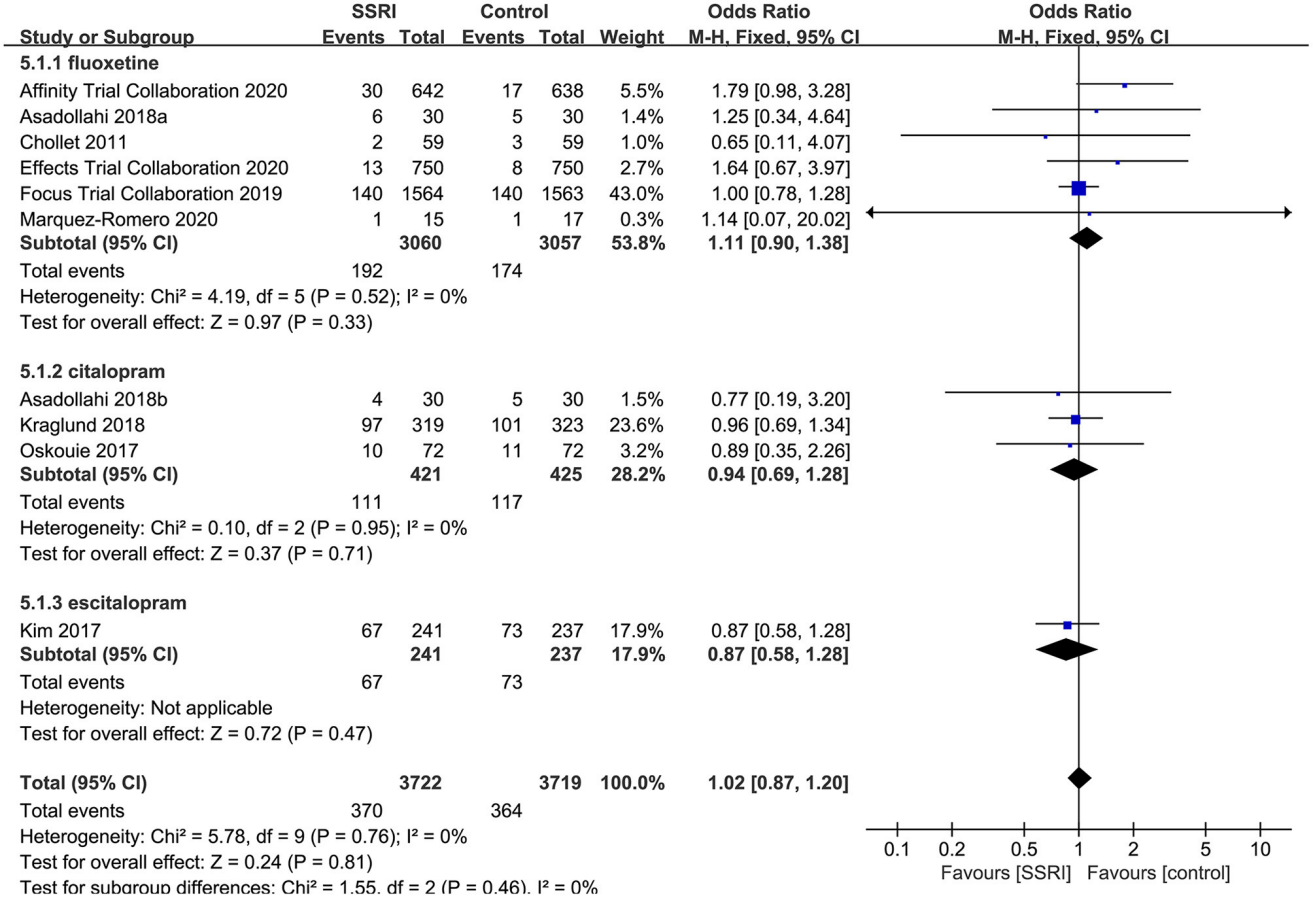
Forest plot of total withdrawal rate.

All RCTs described total withdrawal rate, the results showed that the total withdrawal rate of fluoxetine (6RCTs, OR = 1.11, 95% CI 0.90–1.27, *P* = 1.38), citalopram (3RCTs, OR = 0.94, 95% CI 0.69–1.28, *P* = 0.71) and escitalopram (1RCT, OR = 0.87, 95% CI 0.58–1.28, *P* = 0.47) were not significantly different compared with placebo. No heterogeneity was detected (*P* = 0.76, *I*^2^ = 0%) (Figure 6).

##### The Incidence of AEs

**Figure 7:**
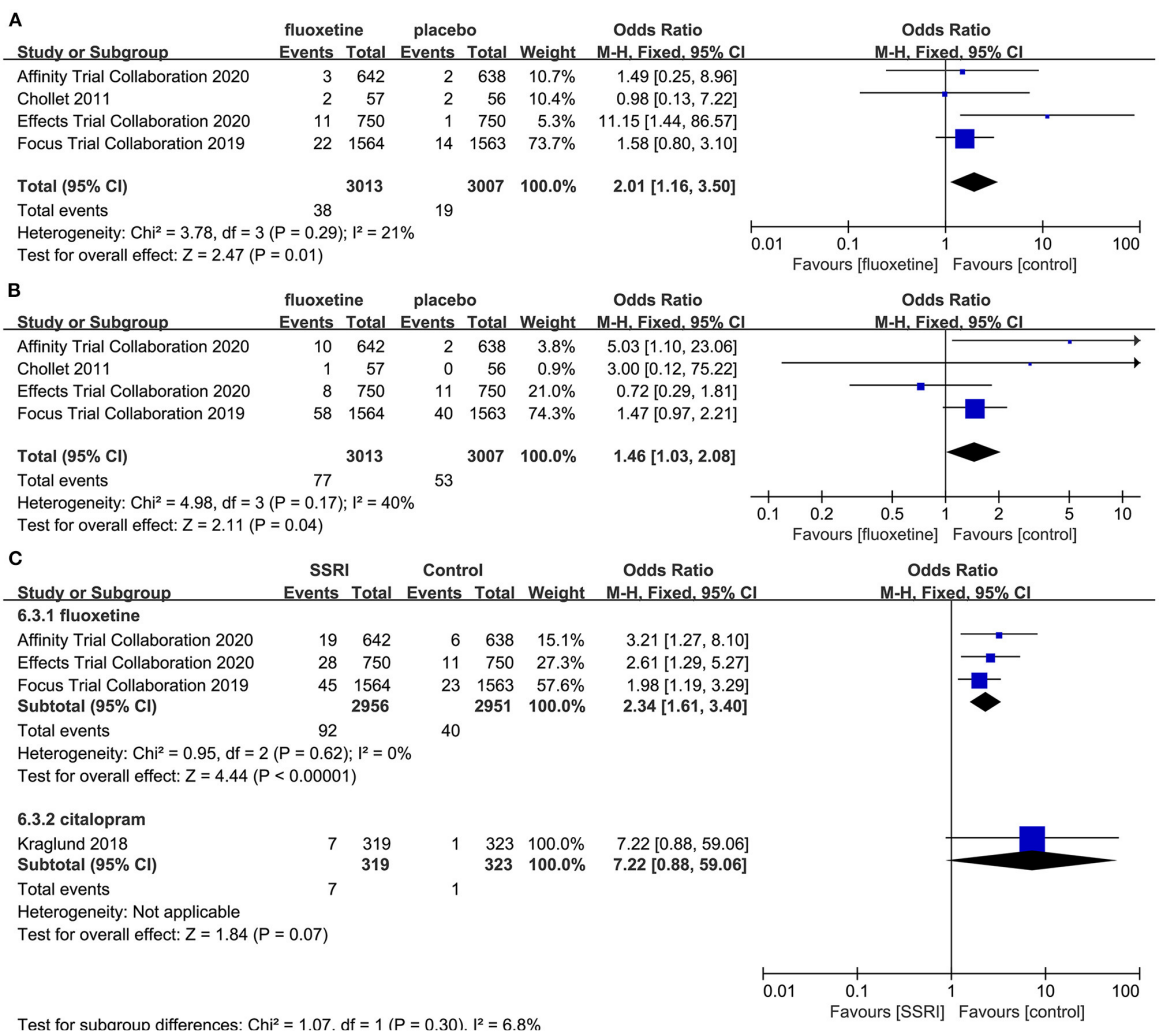
Forest plot of adverse events. **(A)** hyponatremia, **(B)** seizure, **(C)** fracture.

After the meta-analysis of the AEs reported in the included RCTs, we found that the incidence of hyponatremia (4–6, 8] (OR = 2.01, 95% CI 1.16–3.50, *P* = 0.01) (Figure 7A), seizure (4–6, 8] (OR = 1.46, 95% CI 1.03–2.08, *P* = 0.04) (Figure 7B), and fracture (5, 6, 8, 23] (OR = 2.34, 95% CI 1.61–3.40, *P* < 0.00001) (Figure 7C) in the fluoxetine group was higher than in the placebo group. Other AEs unrelated to SSRIs reported in the included RCTs were shown in Table 2.

**Table 2:** Meta-analyses of AEs not related to SSRIs reported in the included RCTs.

**AEs**	**RCTs (N)**	**OR [95% CI]**	**Heterogeneity**	**Effect model**	**Overall effect (*P*-value)**
Attempted or actual suicide	3	0.82 [0.23–2.84]	*P =* 0.52, *I*^2^ *=* 0%	Fixed	*P =* 0.75
Fall with injury	2	1.74 [0.82–3.71]	*P =* 0.08, *I*^2^ *=* 66%	Random	*P =* 0.15
Upper gastrointestinal bleed	3	1.30 [0.72–2.34]	*P =* 0.98, *I*^2^ *=* 0%	Fixed	*P =* 0.38
Haemorrhagic stroke	4	1.13 [0.56–2.27]	*P =* 0.47, *I*^2^ *=* 0%	Fixed	*P =* 0.73
All bleeding events	4	1.18 [0.84–1.65]	*P =* 0.84, *I*^2^ *=* 0%	Fixed	*P =* 0.34
Acute coronary events	4	0.61 [0.36–1.01]	*P =* 0.79, *I*^2^ *=* 0%	Fixed	*P =* 0.06
Ischaemic stroke	4	0.93 [0.69–1.25]	*P =* 0.28, *I*^2^ *=* 21%	Fixed	*P =* 0.64
All thrombotic events	3	0.76 [0.58–1.00]	*P =* 0.42, *I*^2^ *=* 0%	Fixed	*P =* 0.05
Diarrhea	4	1.83 [0.90–3.71]	*P =* 0.48, *I*^2^ *=* 0%	Fixed	*P =* 0.09
Any Stroke	3	0.93 [0.71–1.21]	*P =* 0.24, *I*^2^ *=* 30%	Fixed	*P =* 0.58
Insomnia	6	0.96 [0.60–1.52]	*P =* 0.81, *I*^2^ *=* 0%	Fixed	*P =* 0.86
Abnormal liver function	2	0.54 [0.25–1.17]	*P =* 0.63, *I*^2^ *=* 0%	Fixed	*P =* 0.12
Dizziness	3	1.07 [0.47–2.46]	*P =* 0.38, *I*^2^ *=* 0%	Fixed	*P =* 0.87
Headache	2	4.23 [0.46–39.26]	*P =* 0.84, *I*^2^ *=* 0%	Fixed	*P =* 0.20
Sweating	3	5.29 [0.90–31.15]	*P =* 0.91, *I^2^ =* 0%	Fixed	*P =* 0.07
Increased appetite	2	3.90 [0.77–19.79]	*P =* 0.88, *I^2^ =* 0%	Fixed	*P =* 0.10
Palpitation	3	0.97 [0.13–6.99]	*P =* 0.34, *I^2^ =* 0%	Fixed	*P =* 0.97
Abdominal pain	2	1.73 [0.50–6.00]	*P =* 0.49, *I^2^ =* 0%	Fixed	*P =* 0.39
Death	8	0.99 [0.80–1.22]	*P =* 0.58, *I^2^ =* 0%	Fixed	*P =* 0.91
Drowsiness	3	1.55 [0.74–3.27]	*P =* 0.94, *I^2^ =* 0%	Fixed	*P =* 0.25
Fatigue	2	0.98 [0.37–2.60]	*P =* 0.52, *I^2^ =* 0%	Fixed	*P =* 0.97
Sexual Dysfunction	3	1.71 [0.73–4.03]	*P =* 0.73, *I^2^ =* 0%	Fixed	*P =* 0.22
Restlessness	2	3.00 [0.12–77.17]	–	Fixed	*P =* 0.51
Hyperglycaemia	3	0.73 [0.17–3.10]	*P =* 0.02, *I^2^ =* 83%	Random	*P* = 0.67
Symptomatic hypoglycaemia	2	1.78 [0.90–3.53]	–	Fixed	*P* = 0.10

*AEs, adverse events; SSRIs, selective serotonin reuptake inhibitors; RCTs, randomized controlled trials; OR, odds ratios; CI, confidence intervals*.

### Publication Bias

**Figure 8:**
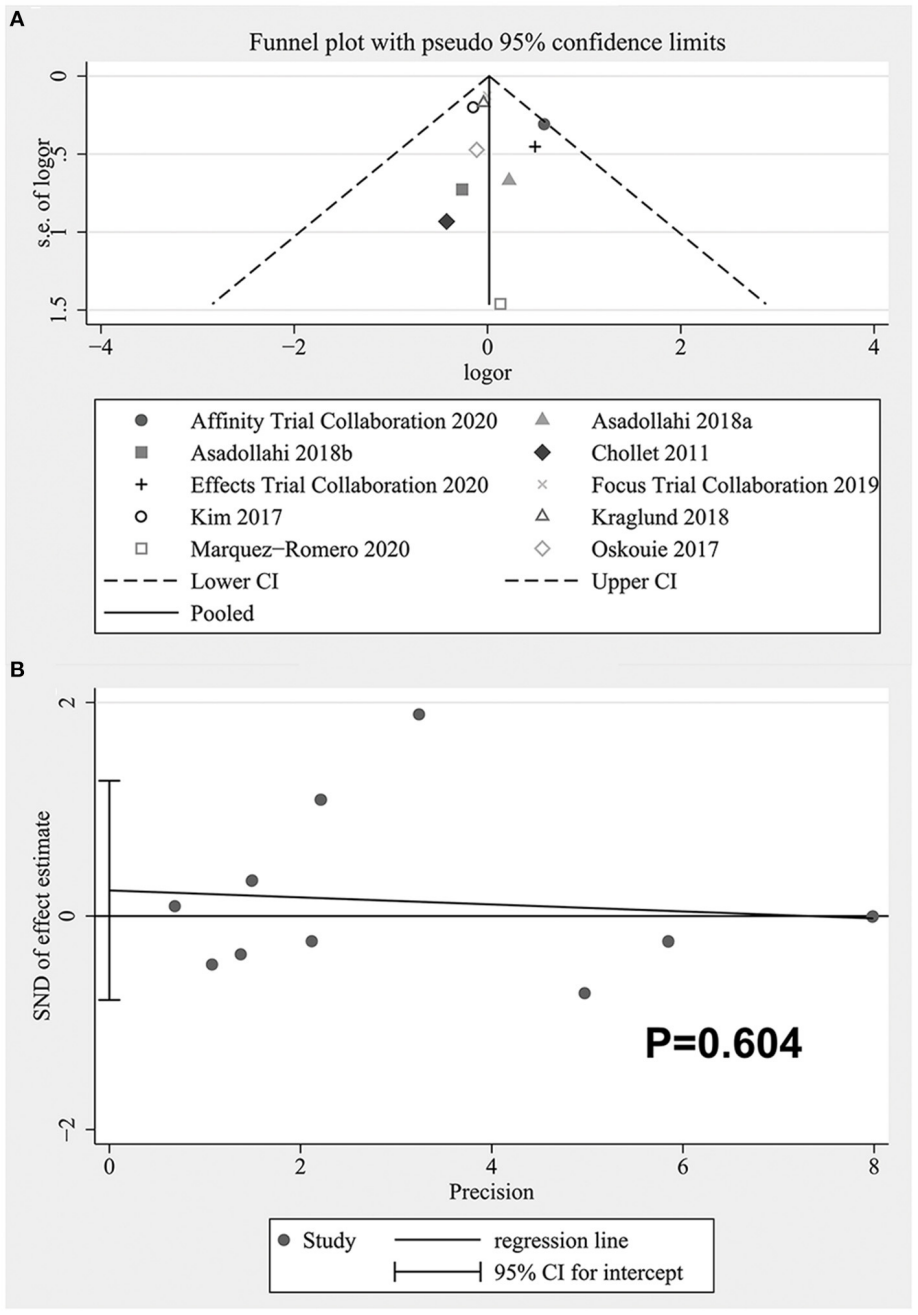
**(A)** The funnel plot of publication bias, **(B)** Egger's test of publication bias.

The funnel plot was shown in Figure 8A. It could be seen that there might be publication bias and the asymmetry was mainly caused by the existence of small sample studies (23), but the quantitative analysis did not support the existence of publication bias (Begg's: *P* = 0.721, Egger's: *P* = 0.604). See Figure 8B for the Egger test diagram.

### Sensitivity Analyses

We conducted sensitivity analyses on the main outcome indicators and the results were all robust (see Supplementary File 2).

## Discussion

### Summary of Main Results

In this meta-analysis, we included 9 trials (10RCTs), including 7,411 participants, 3,722 participants in the experimental group, and 3,689 participants in the control group. In terms of efficacy, the results showed that fluoxetine and citalopram improved the FMMS score better than the placebo. However, there was no significant difference in NIHSS, mRS and BI score between the two groups. Concerning the tolerability, there was no evident difference in the total withdrawal rate between the two groups. After summarizing and analyzing a variety of AEs reported in the RCTs, we found that the incidence of hyponatremia, seizure, and fracture in the fluoxetine group was higher than that in the placebo group, and there was no statistical difference in the incidence of other reported AEs between the two groups.

### Overall Completeness and Applicability of Evidence

In electronic database retrieval, according to our relatively broad search strategy, we could obtain as much literature as possible about SSRIs and stroke, to avoid missing eligible literature as far as possible. On this basis, we then screened the trials that met our inclusion criteria. We also searched unpublished studies and references that may meet the inclusion criteria, Besides, all the RCTs included fully reported predetermined results and provided detailed data for analysis. Thus, the comprehensiveness of our meta-analysis could be realized.

The trials included in the meta-analysis are all double-blind, placebo-controlled RCTs, and a total of 7,411 participants are included in the analysis. The sample size is large enough, and patients with ischemic and hemorrhagic stroke are also included in the study. Therefore, we believe that the basic characteristics of patients can represent the current situation of patients in the real world, so we consider that the results of the meta-analysis are applicable to clinical practice. However, most of the RCTs exclude patients with cognitive impairment, aphasia, and severe strokes that could be life-threatening, which may be a negative factor affecting the applicability of evidence, as such patients also account for a certain proportion in actual clinical practice. Patients enrolled in all RCTs have different time requirements from the onset, but the maximum time is within 3 weeks of onset, and since only fluoxetine, citalopram, and escitalopram are involved in the study, we can not provide evidence on the efficacy and tolerability of SSRIs beyond 3 weeks or other SSRIs.

### Quality of the Evidence

We have evaluated the study based on the information provided in the RCTs, and the overall risk of bias is low. Among them, the baseline data provided by Kim et al. and Savadi Oskouie et al. (22, 24) is not the actual baseline values, so attrition bias and other bias may exist. In addition, a few RCTs do not report in detail the allocation concealment and blind method, making it difficult to determine the risk of bias. However, in the final pooled analysis of the main results, the heterogeneity is low, indicating that the included RCTs have good homogeneity, so the combined results are reliable.

### Potential Biases in the Review Process

In the process of review, two authors conducted the review separately, and when the results were inconsistent, the decision was made after consultation with the third author, which was less likely to make mistakes than the repeated review by a single author twice. However, there might still be some limitations. On the one hand, only fluoxetine, citalopram, and escitalopram are included in our meta-analysis, so we can not draw conclusions about other SSRIs that are not included. On the other hand, we only include studies published in English, which might result in missing some high-quality, large-sample RCTs published in other languages.

### Agreements and Disagreements With Other Studies or Reviews

Our meta-analysis focuses on the motor recovery after acute stroke in non-depressed patients treated with SSRIs, and the consistency and inconsistency with previously published studies are as follows.

Regarding efficacy, our results showed that fluoxetine and citalopram improved FMMS score but not NIHSS, BI, and mRS score, which is consistent with the results of Zhou et al. (25), while Mead et al. (7) showed SSRIs improved dependence, disability, and neurological impairment, which is inconsistent with our results. However, due to the inclusion of patients with post-stroke depression in the study, the incomplete use of a matching placebo in the control group, and, most importantly, the high heterogeneity and methodological limitations between the studies, thus the results are less reliable. The results of subsequent Legg et al. (9) are basically consistent with ours, which only included low risk of bias RCTs, and concluded that SSRIs could not improve dependence, disability, and neurological impairment, but the number of included RCTs and the effect size is small.

Regarding AEs, we found that fluoxetine was associated with hyponatremia, seizure, and fracture, but Mead et al., Legg et al., and Jones et al. (7, 9, 26) found no significant difference in the incidence of seizure between the two groups. The reason why the results are inconsistent is that the studies included are different. Zhou et al. (25) showed that SSRIs increased the incidence of nausea and seizure, and decreased the incidence of psychiatric disorders. The findings of seizure are consistent with ours, but we found no evidence for nausea or psychiatric disorders. Jones et al. (26) showed that SSRIs could increase the risk of fracture and the studies included were the same as ours. But considering that fluoxetine and citalopram were involved, we made an analysis based on the type of drugs. Since there was only one study of citalopram, we could not conclude that citalopram increased the risk of fracture. Other AEs not associated with SSRIs, such as falls and recurrent stroke, are consistent with our study. Besides, the data for hyponatremia is not mentioned in these four studies.

## Conclusions

### Implications for Practice

Our meta-analysis can not provide sufficient evidence for the routine use of SSRIs in patients not clinically diagnosed with depression following acute stroke. Current results suggested that fluoxetine and citalopram could improve FMMS scores in patients with acute stroke, but NIHSS, BI, and mRS scores were not significantly improved, and data on the motor components of NIHSS scores were unavailable. The difference in the evaluation emphasis of each scale may be a factor for the difference in the results. In addition, patients with acute stroke accompanied by aphasia and cognitive impairment are also common in clinical work, and there is no relevant evidence for these patients. And that fluoxetine may increase the risk of hyponatremia, seizure, and fracture, so the prevention of these possible AEs should be worth our attention. Therefore, in clinical practice, it is necessary to carefully evaluate and weigh the advantages and disadvantages before deciding whether to use SSRIs in combination with the actual situation of patients. If we finally decide to initiate an SSRI, we should consider targeted prevention of these AEs to maximize the benefits.

### Implications for Research

Fluoxetine is the most commonly used drug in RCTs included in our meta-analysis, and it is also currently used in several large-scale RCTs, so it could be the first choice for future studies. However, there is still a lack of data on whether other drugs other than fluoxetine improve motor recovery after acute stroke, thus we look forward to further large RCTs to supplement the existing evidence. Future studies should also focus on the occurrence of AEs and include patients with aphasia and cognitive impairment so that the results can be more applicable to clinical practice. We also look forward to future network meta-analyses to select the antidepressant with the best efficacy and tolerability when data are available.

## Data Availability Statement

The original contributions presented in the study are included in the article/Supplementary Material, further inquiries can be directed to the corresponding author/s.

## Author Contributions

CWa and NS contributed conception and design of the study. YY and NS organized the database. CWe and NS performed the statistical analysis. CWa wrote the first draft of the manuscript. NS wrote sections of the manuscript. SG and CWe revised and proofread the manuscript. All authors contributed to the article and approved the submitted version.

## Conflict of Interest

The authors declare that the research was conducted in the absence of any commercial or financial relationships that could be construed as a potential conflict of interest.

## Publisher's Note

All claims expressed in this article are solely those of the authors and do not necessarily represent those of their affiliated organizations, or those of the publisher, the editors and the reviewers. Any product that may be evaluated in this article, or claim that may be made by its manufacturer, is not guaranteed or endorsed by the publisher.
