# Supplementary material for: The Efficacy and Tolerability of Selective Serotonin Reuptake Inhibitors for Motor Recovery in Non-depressed Patients After Acute Stroke: A Meta-Analysis
Source: Front Neurol. 2021 Oct 20;12:749322. doi: 10.3389/fneur.2021.749322 (PMC8564176; doi:10.3389/fneur.2021.749322)
Supplement: Supplementary file 1 [file Table_1.DOCX]

**Supplementary S1 file.** Detailed Search Strategy

Search strategy in PubMed

#1 stroke [Mesh]——137611

#2 (Strokes OR "Cerebrovascular Accident" OR "Cerebrovascular Accidents" OR "CVA(Cerebrovascular Accident)" OR CVAs OR " Cerebrovascular Apoplexy" OR "Apoplexy, Cerebrovascular" OR "Vascular Accident, Brain" OR "Brain Vascular Accident" OR "Brain Vascular Accidents" OR "Vascular Accidents, Brain" OR "Cerebrovascular Stroke" OR "Cerebrovascular Strokes" OR "Stroke, Cerebrovascular" OR "Strokes, Cerebrovascular" OR Apoplexy OR "Cerebral Stroke" OR "Cerebral Strokes" OR "Stroke, Cerebral" OR " Strokes, Cerebral" OR "Stroke, Acute" OR "Acute Stroke" OR "Acute Strokes" OR "Strokes, Acute" OR "Cerebrovascular Accident, Acute" OR "Acute Cerebrovascular Accident" OR "Acute Cerebrovascular Accidents" OR "Cerebrovascular Accidents, Acute") [Title/Abstract]——361236

#3 "cerebral infarction" [Mesh]——32608

#4 ("Cerebral Infarctions" OR "Infarctions, Cerebral" OR "Infarction, Cerebral" OR "Cerebral Infarct" OR "Cerebral Infarcts" OR "Infarct, Cerebral" OR "Infarcts, Cerebral" OR "Cerebral Infarction, Left Hemisphere" OR "Left Hemisphere, Infarction, Cerebral" OR "Infarction, Left Hemisphere, Cerebral" OR "Left Hemisphere, Cerebral Infarction" OR "Cerebral, Left Hemisphere, Infarction" OR "Infarction, Cerebral, Left Hemisphere" OR "Subcortical Infarction" OR "Infarction, Subcortical" OR "Infarctions, Subcortical" OR "Subcortical Infarctions" OR "Posterior Choroidal Artery Infarction" OR "Anterior Choroidal Artery Infarction" OR "Cerebral Infarction, Right Hemisphere" OR "Right Hemisphere, Cerebral Infarction" OR "Infarction, Right Hemisphere, Cerebral" OR "Infarction, Cerebral, Right Hemisphere" OR "Right Hemisphere, Infarction, Cerebral" OR "Cerebral, Right Hemisphere, Infarction")[Title/Abstract]——6077

#5 "brain infarction"[Mesh]——38428

#6 ("Brain Infarctions" OR "Infarction, Brain" OR "Infarctions, Brain" OR "Brain Infarct" OR "Brain Infarcts" OR "Infarct, Brain" OR "Infarcts, Brain" OR "Anterior Circulation Brain Infarction" OR "Infarction, Brain, Anterior Circulation" OR "Infarction, Anterior Circulation, Brain" OR "Anterior Circulation Infarction, Brain" OR "Brain Infarction, Anterior Circulation" OR "Venous Infarction, Brain" OR "Brain Venous Infarction" OR "Brain Venous Infarctions" OR "Infarction, Brain Venous" OR "Infarctions, Brain Venous" OR " Venous Infarctions, Brain" OR "Brain Infarction, Venous" OR "Brain Infarctions, Venous" OR "Infarction, Venous Brain" OR "Infarctions, Venous Brain" OR "Venous Brain Infarction" OR "Venous Brain Infarctions" OR "Anterior Cerebral Circulation Infarction" OR "Infarction, Anterior Cerebral Circulation" OR "Brain Infarction, Posterior Circulation" OR "Posterior Circulation Infarction, Brain" OR "Posterior Circulation Brain Infarction" OR "Infarction, Brain, Posterior Circulation" OR "Infarction, Posterior Circulation, Brain")[Title/Abstract]——3500

#7 "cerebral hemorrhage"[Mesh]——34225

#8 ("Hemorrhage, Cerebrum" OR "Cerebrum Hemorrhage" OR "Cerebrum Hemorrhages" OR "Hemorrhages, Cerebrum" OR "Cerebral Parenchymal Hemorrhage" OR "Cerebral Parenchymal Hemorrhages" OR "Hemorrhage, Cerebral Parenchymal " OR "Hemorrhages, Cerebral Parenchymal" OR "Parenchymal Hemorrhage, Cerebral" OR "Parenchymal Hemorrhages, Cerebral" OR "Intracerebral Hemorrhage" OR "Hemorrhage, Intracerebral" OR "Hemorrhages, Intracerebral" OR "Intracerebral Hemorrhages" OR "Hemorrhage, Cerebral" OR "Cerebral Hemorrhages" OR "Hemorrhages, Cerebral" OR "Brain Hemorrhage, Cerebral" OR "Brain Hemorrhages, Cerebral" OR "Cerebral Brain Hemorrhage" OR "Cerebral Brain Hemorrhages" OR "Hemorrhage, Cerebral Brain" OR "Hemorrhages, Cerebral Brain")[Title/Abstract]——17176

#9 "Intracranial Hemorrhages"[Mesh]——71979

#10 ("Hemorrhages, Intracranial" OR "Intracranial Hemorrhage" OR "Hemorrhage, Intracranial" OR "Posterior Fossa Hemorrhage" OR "Hemorrhage, Posterior Fossa" OR "Hemorrhages, Posterior Fossa" OR "Posterior Fossa Hemorrhages" OR "Brain Hemorrhage" OR "Brain Hemorrhages" OR "Hemorrhage, Brain" OR "Hemorrhages, Brain")[Title/Abstract]——13338

#11 #1 OR #2 OR #3 OR #4 OR #5 OR #6 OR #7 OR #8 OR #9 OR #10——426478

#12 "Serotonin Uptake Inhibitors"[Mesh]——19604

#13 ("5-Hydroxytryptamine Uptake Inhibitors" OR "5 Hydroxytryptamine Uptake Inhibitors" OR "Inhibitors, 5-HT Uptake" OR "Inhibitors, 5 HT Uptake" OR "Inhibitors, 5-Hydroxytryptamine Uptake" OR "Inhibitors, 5 Hydroxytryptamine Uptake" OR "Inhibitors, Serotonin Reuptake" OR "Reuptake Inhibitors, Serotonin" OR "Serotonin Reuptake Inhibitors" OR "Uptake Inhibitors, 5-HT" OR "Uptake Inhibitors, 5 HT" OR "Uptake Inhibitors, 5-Hydroxytryptamine" OR "Uptake Inhibitors, 5 Hydroxytryptamine" OR "Uptake Inhibitors, Serotonin" OR "5-HT Uptake Inhibitors" OR "5 HT Uptake Inhibitors" OR "Inhibitors, Serotonin Uptake" OR "Selective Serotonin Reuptake Inhibitors")[Title/Abstract]——10562

#14 Citalopram[Mesh]——4878

#15 (Cytalopram OR Seropram OR Escitalopram OR Celexa OR Lu-10-171 OR Lu10171 OR "Citalopram Hydrobromide" OR "Escitalopram Oxalate" OR Lexapro)[Title/Abstract]——2689

#16 Fluoxetine[Mesh]——9168

#17 (Fluoxetin OR N-Methyl-gamma-(4-(trifluoromethyl)phenoxy)benzenepropanamine OR Lilly-110140 OR "Lilly 110140" OR Lilly110140 OR Sarafem OR "Fluoxetine Hydrochloride" OR Prozac)[Title/Abstract]——722

#18 Paroxetine[Mesh]——3974

#19 (Aropax OR BRL-29060 OR "BRL 29060" OR BRL29060 OR FG-7051 OR "FG 7051" OR FG7051 OR "Paroxetine Acetate" OR Seroxat OR "Paroxetine Hydrochloride Anhydrous" OR "Paroxetine Maleate" OR "Paroxetine, cis-(+)-Isomer" OR "Paroxetine, cis-(-)-Isomer" OR "Paroxetine, trans-(+)-Isomer" OR Paxil OR "Paroxetine Hydrochloride Hemihydrate" OR "Paroxetine Hydrochloride, Hemihydrate" OR "Paroxetine Hydrochloride")[Title/Abstract]——234

#20 Sertraline[Mesh]——3070

#21 (Zoloft OR Altruline OR Lustral OR Apo-Sertraline OR "Apo Sertraline" OR Aremis OR Besitran OR Sealdin OR Gladem OR Novo-Sertraline OR "Novo Sertraline" OR ratio-Sertraline OR "ratio Sertraline" OR Rhoxal-sertraline OR "Rhoxal sertraline" OR "Sertraline Hydrochloride" OR "Hydrochloride, Sertraline" OR "Sertraline Hydrochloride (1S-cis)-Isomer" OR Gen-Sertraline OR "Gen Sertraline")[Title/Abstract]——4666

#22 Fluvoxamine[Mesh]——1862

#23 (Fluvoxadura OR "Fluvoxamin AL" OR "Fluvoxamin beta" OR "Fluvoxamin Stada" OR Fluvoxamin-neuraxpharm OR "Fluvoxamin neuraxpharm" OR Fluvoxamin-ratiopharm OR "Fluvoxamin ratiopharm" OR ratio-Fluvoxamine OR "ratio Fluvoxamine" OR "Fluvoxamina Geminis" OR "Geminis, Fluvoxamina" OR "Fluvoxamine Maleate" OR "Fluvoxamine Maleate, (E)-Isomer" OR "Fluvoxamine, (Z)-Isomer" OR Luvox OR Floxyfral OR Fevarin OR Dumirox OR Faverin OR Novo-Fluvoxamine OR "Novo Fluvoxamine" OR Nu-Fluvoxamine OR "Nu Fluvoxamine" OR PMS-Fluvoxamine OR "PMS Fluvoxamine" OR Desiflu OR DU-23000 OR "DU 23000" OR DU23000) [Title/Abstract]——235

#24 #12 OR #13 OR #14 OR #15 OR #16 OR #17 OR #18 OR #19 OR #20 OR #21 OR #22 OR #23——37716

#25 #11 AND #24 AND (Filters applied: Randomized Controlled Trial, Humans, English)——79

Search strategy in Web of Science

#1 (TS=(Stroke OR Strokes OR "Cerebrovascular Accident" OR "Cerebrovascular Accidents" OR "CVA(Cerebrovascular Accident)" OR CVAs OR " Cerebrovascular Apoplexy" OR "Apoplexy, Cerebrovascular" OR "Vascular Accident, Brain" OR "Brain Vascular Accident" OR "Brain Vascular Accidents" OR "Vascular Accidents, Brain" OR "Cerebrovascular Stroke" OR "Cerebrovascular Strokes" OR "Stroke, Cerebrovascular" OR "Strokes, Cerebrovascular" OR Apoplexy OR "Cerebral Stroke" OR "Cerebral Strokes" OR "Stroke, Cerebral" OR " Strokes, Cerebral" OR "Stroke, Acute" OR "Acute Stroke" OR "Acute Strokes" OR "Strokes, Acute" OR "Cerebrovascular Accident, Acute" OR "Acute Cerebrovascular Accident" OR "Acute Cerebrovascular Accidents" OR "Cerebrovascular Accidents, Acute")) AND language: (English)——352805

#2 (TS=("cerebral infarction" OR "Cerebral Infarctions" OR "Infarctions, Cerebral" OR "Infarction, Cerebral" OR "Cerebral Infarct" OR "Cerebral Infarcts" OR "Infarct, Cerebral" OR "Infarcts, Cerebral" OR "Cerebral Infarction, Left Hemisphere" OR "Left Hemisphere, Infarction, Cerebral" OR "Infarction, Left Hemisphere, Cerebral" OR "Left Hemisphere, Cerebral Infarction" OR "Cerebral, Left Hemisphere, Infarction" OR "Infarction, Cerebral, Left Hemisphere" OR "Subcortical Infarction" OR "Infarction, Subcortical" OR "Infarctions, Subcortical" OR "Subcortical Infarctions" OR "Posterior Choroidal Artery Infarction" OR "Anterior Choroidal Artery Infarction" OR "Cerebral Infarction, Right Hemisphere" OR "Right Hemisphere, Cerebral Infarction" OR "Infarction, Right Hemisphere, Cerebral" OR "Infarction, Cerebral, Right Hemisphere" OR "Right Hemisphere, Infarction, Cerebral" OR "Cerebral, Right Hemisphere, Infarction")) AND language: (English)——19592

#3 (TS=("brain infarction" OR "Brain Infarctions" OR "Infarction, Brain" OR "Infarctions, Brain" OR "Brain Infarct" OR "Brain Infarcts" OR "Infarct, Brain" OR "Infarcts, Brain" OR "Anterior Circulation Brain Infarction" OR "Infarction, Brain, Anterior Circulation" OR "Infarction, Anterior Circulation, Brain" OR "Anterior Circulation Infarction, Brain" OR "Brain Infarction, Anterior Circulation" OR "Venous Infarction, Brain" OR "Brain Venous Infarction" OR "Brain Venous Infarctions" OR "Infarction, Brain Venous" OR "Infarctions, Brain Venous" OR " Venous Infarctions, Brain" OR "Brain Infarction, Venous" OR "Brain Infarctions, Venous" OR "Infarction, Venous Brain" OR "Infarctions, Venous Brain" OR "Venous Brain Infarction" OR "Venous Brain Infarctions" OR "Anterior Cerebral Circulation Infarction" OR "Infarction, Anterior Cerebral Circulation" OR "Brain Infarction, Posterior Circulation" OR "Posterior Circulation Infarction, Brain" OR "Posterior Circulation Brain Infarction" OR "Infarction, Brain, Posterior Circulation" OR "Infarction, Posterior Circulation, Brain")) AND language: (English)——5350

#4 (TS=("cerebral hemorrhage" OR "Hemorrhage, Cerebrum" OR "Cerebrum Hemorrhage" OR "Cerebrum Hemorrhages" OR "Hemorrhages, Cerebrum" OR "Cerebral Parenchymal Hemorrhage" OR "Cerebral Parenchymal Hemorrhages" OR "Hemorrhage, Cerebral Parenchymal " OR "Hemorrhages, Cerebral Parenchymal" OR "Parenchymal Hemorrhage, Cerebral" OR "Parenchymal Hemorrhages, Cerebral" OR "Intracerebral Hemorrhage" OR "Hemorrhage, Intracerebral" OR "Hemorrhages, Intracerebral" OR "Intracerebral Hemorrhages" OR "Hemorrhage, Cerebral" OR "Cerebral Hemorrhages" OR "Hemorrhages, Cerebral" OR "Brain Hemorrhage, Cerebral" OR "Brain Hemorrhages, Cerebral" OR "Cerebral Brain Hemorrhage" OR "Cerebral Brain Hemorrhages" OR "Hemorrhage, Cerebral Brain" OR "Hemorrhages, Cerebral Brain")) AND language: (English)——22366

#5 (TS=("Intracranial Hemorrhages" OR "Hemorrhages, Intracranial" OR "Intracranial Hemorrhage" OR "Hemorrhage, Intracranial" OR "Posterior Fossa Hemorrhage" OR "Hemorrhage, Posterior Fossa" OR "Hemorrhages, Posterior Fossa" OR "Posterior Fossa Hemorrhages" OR "Brain Hemorrhage" OR "Brain Hemorrhages" OR "Hemorrhage, Brain" OR "Hemorrhages, Brain")) AND language: (English)——12891

#6 #1 OR #2 OR #3 OR #4 OR #5——380461

#7 (TS=("Serotonin Uptake Inhibitors" OR "5-Hydroxytryptamine Uptake Inhibitors" OR "5 Hydroxytryptamine Uptake Inhibitors" OR "Inhibitors, 5-HT Uptake" OR "Inhibitors, 5 HT Uptake" OR "Inhibitors, 5-Hydroxytryptamine Uptake" OR "Inhibitors, 5 Hydroxytryptamine Uptake" OR "Inhibitors, Serotonin Reuptake" OR "Reuptake Inhibitors, Serotonin" OR "Serotonin Reuptake Inhibitors" OR "Uptake Inhibitors, 5-HT" OR "Uptake Inhibitors, 5 HT" OR "Uptake Inhibitors, 5-Hydroxytryptamine" OR "Uptake Inhibitors, 5 Hydroxytryptamine" OR "Uptake Inhibitors, Serotonin" OR "5-HT Uptake Inhibitors" OR "5 HT Uptake Inhibitors" OR "Inhibitors, Serotonin Uptake" OR "Selective Serotonin Reuptake Inhibitors")) AND language: (English)——14305

#8 (TS=(Citalopram OR Cytalopram OR Seropram OR Escitalopram OR Celexa OR Lu-10-171 OR Lu10171 OR "Citalopram Hydrobromide" OR "Escitalopram Oxalate" OR Lexapro)) AND language: (English)——9013

#9 (TS=(Fluoxetine OR Fluoxetin OR N-Methyl-gamma-(4-(trifluoromethyl)phenoxy)benzenepropanamine OR Lilly-110140 OR "Lilly 110140" OR Lilly110140 OR Sarafem OR "Fluoxetine Hydrochloride" OR Prozac)) AND language: (English)——19698

#10 (TS=(Paroxetine OR Aropax OR BRL-29060 OR "BRL 29060" OR BRL29060 OR FG-7051 OR "FG 7051" OR FG7051 OR "Paroxetine Acetate" OR Seroxat OR "Paroxetine Hydrochloride Anhydrous" OR "Paroxetine Maleate" OR "Paroxetine, cis-(+)-Isomer" OR "Paroxetine, cis-(-)-Isomer" OR "Paroxetine, trans-(+)-Isomer" OR Paxil OR "Paroxetine Hydrochloride Hemihydrate" OR "Paroxetine Hydrochloride, Hemihydrate" OR "Paroxetine Hydrochloride")) AND language: (English)——8327

#11 (TS=(Sertraline OR Zoloft OR Altruline OR Lustral OR Apo-Sertraline OR "Apo Sertraline" OR Aremis OR Besitran OR Sealdin OR Gladem OR Novo-Sertraline OR "Novo Sertraline" OR ratio-Sertraline OR "ratio Sertraline" OR Rhoxal-sertraline OR "Rhoxal sertraline" OR "Sertraline Hydrochloride" OR "Hydrochloride, Sertraline" OR "Sertraline Hydrochloride (1S-cis)-Isomer" OR Gen-Sertraline OR "Gen Sertraline")) AND language: (English)——6689

#12 (TS=(Fluvoxamine OR Fluvoxadura OR "Fluvoxamin AL" OR "Fluvoxamin beta" OR "Fluvoxamin Stada" OR Fluvoxamin-neuraxpharm OR "Fluvoxamin neuraxpharm" OR Fluvoxamin-ratiopharm OR "Fluvoxamin ratiopharm" OR ratio-Fluvoxamine OR "ratio Fluvoxamine" OR "Fluvoxamina Geminis" OR "Geminis, Fluvoxamina" OR "Fluvoxamine Maleate" OR "Fluvoxamine Maleate, (E)-Isomer" OR "Fluvoxamine, (Z)-Isomer" OR Luvox OR Floxyfral OR Fevarin OR Dumirox OR Faverin OR Novo-Fluvoxamine OR "Novo Fluvoxamine" OR Nu-Fluvoxamine OR "Nu Fluvoxamine" OR PMS-Fluvoxamine OR "PMS Fluvoxamine" OR Desiflu OR DU-23000 OR "DU 23000" OR DU23000)) AND language: (English)——3894

#13 #7 OR #8 OR #9 OR #10 OR #11 OR #12——44873

#14 #6 AND #13 AND publication type:(ARTICLE)——520

Search strategy in Embase

#1 ('cerebrovascular accident'/exp/dm_dt OR 'cerebrovascular accident') AND [randomized controlled trial]/lim AND ([article]/lim OR [article in press]/lim) AND [humans]/lim AND [english]/lim AND [embase]/lim——8040

#2 ('accident, cerebrovascular':ab,ti OR 'acute cerebrovascular lesion':ab,ti OR 'acute focal cerebral vasculopathy':ab,ti OR 'acute stroke':ab,ti OR 'apoplectic stroke':ab,ti OR apoplexia:ab,ti OR apoplexy:ab,ti OR 'blood flow disturbance, brain':ab,ti OR 'brain accident':ab,ti OR 'brain attack':ab,ti OR 'brain blood flow disturbance':ab,ti OR 'brain insult':ab,ti OR 'brain insultus':ab,ti OR 'brain ischaemic attack':ab,ti OR 'brain ischemic attack':ab,ti OR 'brain vascular accident':ab,ti OR 'cerebral apoplexia':ab,ti OR 'cerebral insult':ab,ti OR 'cerebral stroke':ab,ti OR 'cerebral vascular accident':ab,ti OR 'cerebral vascular insufficiency':ab,ti OR 'cerebro vascular accident':ab,ti OR 'cerebrovascular arrest':ab,ti OR 'cerebrovascular failure':ab,ti OR 'cerebrovascular injury':ab,ti OR 'cerebrovascular insufficiency':ab,ti OR 'cerebrovascular insult':ab,ti OR 'cerebrum vascular accident':ab,ti OR 'cryptogenic stroke':ab,ti OR cva:ab,ti OR 'ischaemic cerebral attack':ab,ti OR 'ischaemic seizure':ab,ti OR 'ischemic cerebral attack':ab,ti OR 'ischemic seizure':ab,ti OR stroke:ab,ti) AND [randomized controlled trial]/lim AND ([article]/lim OR [article in press]/lim) AND [humans]/lim AND [english]/lim AND [embase]/lim——9291

#3 ('brain infarction'/exp/dm_dt OR 'brain infarction') AND [randomized controlled trial]/lim AND ([article]/lim OR [article in press]/lim) AND [humans]/lim AND [english]/lim AND [embase]/lim——896

#4 ('brain infarct':ab,ti OR 'cerebral infarct':ab,ti OR 'cerebral infarction':ab,ti OR 'cerebrovascular infarction':ab,ti OR 'cortical infarction':ab,ti OR 'hemisphere infarct':ab,ti OR 'hemisphere infarction':ab,ti OR 'hemispheric infarct':ab,ti OR 'hemispheric infarction':ab,ti OR 'infarction, brain':ab,ti OR 'silent brain infarction':ab,ti) AND [randomized controlled trial]/lim AND ([article]/lim OR [article in press]/lim) AND [humans]/lim AND [english]/lim AND [embase]/lim——338

#5 ('brain hemorrhage'/exp/dm_dt OR 'brain hemorrhage') AND [randomized controlled trial]/lim AND ([article]/lim OR [article in press]/lim) AND [humans]/lim AND [english]/lim AND [embase]/lim——2703

#6 ('bleeding, corpus callosum':ab,ti OR 'brain bleeding':ab,ti OR 'brain haemorrhage':ab,ti OR 'brain haemorrhage, traumatic':ab,ti OR 'brain hemorrhage, traumatic':ab,ti OR 'brain microhaemorrhage':ab,ti OR 'brain microhemorrhage':ab,ti OR 'brain stem haemorrhage, traumatic':ab,ti OR 'brain stem hemorrhage, traumatic':ab,ti OR 'cerebral haemorrhage':ab,ti OR 'cerebral haemorrhage, traumatic':ab,ti OR 'cerebral hemorrhage':ab,ti OR 'cerebral hemorrhage, traumatic':ab,ti OR 'cerebral microbleed':ab,ti OR 'corpus callosum bleeding':ab,ti OR 'corpus callosum haemorrhage':ab,ti OR 'corpus callosum hemorrhage':ab,ti OR encephalorrhagia:ab,ti OR 'haemorrhage, brain':ab,ti OR 'haemorrhage, intracranial':ab,ti OR 'haemorrhagic apoplexy':ab,ti OR 'haemorrhagic stroke':ab,ti OR 'haemorrhagic stroke intracerebral bleeding':ab,ti OR hematencephalon:ab,ti OR 'hemorrhage, brain':ab,ti OR 'hemorrhage, intracranial':ab,ti OR 'hemorrhagic apoplexy':ab,ti OR 'hemorrhagic stroke':ab,ti OR 'hemorrhagic stroke intracerebral bleeding':ab,ti OR 'hypertensive intracranial haemorrhage':ab,ti OR 'hypertensive intracranial hemorrhage':ab,ti OR 'intracerebral bleeding':ab,ti OR 'intracerebral haemorrhage':ab,ti OR 'intracerebral hemorrhage':ab,ti OR 'intracortical haemorrhage':ab,ti OR 'intracortical hemorrhage':ab,ti OR 'intracranial bleeding':ab,ti OR 'intracranial haemorrhage':ab,ti OR 'intracranial haemorrhage, hypertensive':ab,ti OR 'intracranial haemorrhage, traumatic':ab,ti OR 'intracranial haemorrhages':ab,ti OR 'intracranial hemorrhage':ab,ti OR 'intracranial hemorrhage, hypertensive':ab,ti OR 'intracranial hemorrhage, traumatic':ab,ti OR 'intracranial hemorrhages':ab,ti OR 'intraventricular haemorrhage':ab,ti OR 'intraventricular hemorrhage':ab,ti OR 'periventricular haemorrhage':ab,ti OR 'periventricular hemorrhage':ab,ti OR 'posterior fossa haemorrhage':ab,ti OR 'posterior fossa hemorrhage':ab,ti OR 'traumatic brain haemorrhage':ab,ti OR 'traumatic brain hemorrhage':ab,ti OR 'traumatic brain stem haemorrhage':ab,ti OR 'traumatic brain stem hemorrhage':ab,ti OR 'traumatic cerebral haemorrhage':ab,ti OR 'traumatic cerebral hemorrhage':ab,ti OR 'traumatic intracranial haemorrhage':ab,ti OR 'traumatic intracranial hemorrhage':ab,ti) AND [randomized controlled trial]/lim AND ([article]/lim OR [article in press]/lim) AND [humans]/lim AND [english]/lim AND [embase]/lim——1411

#7 #1 OR #2 OR #3 OR #4 OR #5 OR #6——13266

#8 ('serotonin uptake inhibitor'/exp OR 'serotonin uptake inhibitor') AND [randomized controlled trial]/lim AND ([article]/lim OR [article in press]/lim) AND [humans]/lim AND [english]/lim AND [embase]/lim——9590

#9 ('antidepressants, serotonin specific reuptake inhibitors':ab,ti OR 'selective serotonin reuptake inhibitor':ab,ti OR 'serotonin reuptake inhibitor':ab,ti OR 'serotonin specific reuptake inhibitor':ab,ti OR 'serotonin specific reuptake inhibitors':ab,ti OR 'serotonin uptake inhibitors':ab,ti OR ssri:ab,ti OR 'ssri antidepressant':ab,ti) AND [randomized controlled trial]/lim AND ([article]/lim OR [article in press]/lim) AND [humans]/lim AND [english]/lim AND [embase]/lim——896

#10 ('citalopram'/exp OR 'citalopram') AND [randomized controlled trial]/lim AND ([article]/lim OR [article in press]/lim) AND [humans]/lim AND [english]/lim AND [embase]/lim——899

#11 ('1 (3 dimethylaminopropyl) 1 (4 fluorophenyl) 1, 3 dihydroisobenzofuran 5 carbonitrile':ab,ti OR '1 (3 dimethylaminopropyl) 1 (4 fluorophenyl) 5 phthalancarbonitrile':ab,ti OR '1 (3 dimethylaminopropyl) 1, 3 dihydro 1 (4 fluorophenyl) isobenzofuran 5 carbonitrile':ab,ti OR '5 phthalancarbonitrile, 1 (3 dimethylaminopropyl) 1 (4 fluorophenyl)':ab,ti OR acelopam:ab,ti OR adeprenal:ab,ti OR 'apo cital':ab,ti OR aurex:ab,ti OR ceform:ab,ti OR celexa:ab,ti OR cilopress:ab,ti OR cinavol:ab,ti OR ciprager:ab,ti OR cipram:ab,ti OR cipramil:ab,ti OR cipraned:ab,ti OR ciprotan:ab,ti OR ciral:ab,ti OR citabax:ab,ti OR citacip:ab,ti OR citagen:ab,ti OR cital:ab,ti OR citalec:ab,ti OR citalich:ab,ti OR citalon:ab,ti OR citalonte:ab,ti OR 'citalopram hydrobromide':ab,ti OR 'citalopram hydrochloride':ab,ti OR 'citalopram ratiopharm':ab,ti OR citalostad:ab,ti OR citalox:ab,ti OR citalvir:ab,ti OR citapram:ab,ti OR citaxin:ab,ti OR citesint:ab,ti OR citopam:ab,ti OR citrol:ab,ti OR citronil:ab,ti OR cytalopram:ab,ti OR dalsan:ab,ti OR elopram:ab,ti OR exenadil:ab,ti OR frimaind:ab,ti OR futuril:ab,ti OR galopran:ab,ti OR humorap:ab,ti OR kaidor:ab,ti OR kitapram:ab,ti OR linisan:ab,ti OR lopracil:ab,ti OR lopraxer:ab,ti OR loxopram:ab,ti OR 'lu 10 171':ab,ti OR 'lu 10171':ab,ti OR 'lu10 171':ab,ti OR lu10171:ab,ti OR lupram:ab,ti OR malicon:ab,ti OR nitalapram:ab,ti OR oropram:ab,ti OR percitale:ab,ti OR pralotam:ab,ti OR 'pram (drug)':ab,ti OR pramital:ab,ti OR prefucet:ab,ti OR pricital:ab,ti OR prisdal:ab,ti OR psiconor:ab,ti OR 'recital (drug)':ab,ti OR renevil:ab,ti OR 'return (citalopram)':ab,ti OR ricap:ab,ti OR ropramin:ab,ti OR selon:ab,ti OR sepram:ab,ti OR seralgan:ab,ti OR seregra:ab,ti OR serital:ab,ti OR seropram:ab,ti OR seror:ab,ti OR sintopram:ab,ti OR sotovon:ab,ti OR talam:ab,ti OR talosin:ab,ti OR 'unstress (drug)':ab,ti OR varom:ab,ti OR vesema:ab,ti OR xadorek:ab,ti OR zanipram:ab,ti OR 'zd 211':ab,ti OR zd211:ab,ti OR zeclicid:ab,ti OR zentius:ab,ti OR zitolex:ab,ti OR zyloram:ab,ti) AND [randomized controlled trial]/lim AND ([article]/lim OR [article in press]/lim) AND [humans]/lim AND [english]/lim AND [embase]/lim——16

#12 ('paroxetine'/exp OR 'paroxetine') AND [randomized controlled trial]/lim AND ([article]/lim OR [article in press]/lim) AND [humans]/lim AND [english]/lim AND [embase]/lim——1168

#13 (ldmp:ti,ab OR '4 (4 fluorophenyl) 3 [ (3, 4 methylenedioxyphenoxy) methyl] piperidine':ti,ab OR 'arketis':ti,ab OR 'aropax':ti,ab OR 'aropax 20':ti,ab OR 'aroxat':ti,ab OR 'brisdelle':ti,ab OR 'brl 29060':ti,ab OR 'brl 29060a':ti,ab OR 'brl29060':ti,ab OR 'brl29060a':ti,ab OR 'daparox':ti,ab OR 'deroxat':ti,ab OR 'dexorat':ti,ab OR 'divarius':ti,ab OR 'dropax':ti,ab OR 'euplix':ti,ab OR 'eutimil':ti,ab OR 'fg 7051':ti,ab OR 'fg7051':ti,ab OR 'frosinor':ti,ab OR 'motivan':ti,ab OR 'optipar':ti,ab OR 'paluxetil':ti,ab OR 'paluxon':ti,ab OR 'paroc':ti,ab OR 'parogen':ti,ab OR 'paroxedura':ti,ab OR 'paroxet':ti,ab OR 'paroxetin':ti,ab OR 'paroxetina':ti,ab OR 'paroxetine':ti,ab OR 'paroxetine hydrochloride':ti,ab OR 'paroxetine mesilate':ti,ab OR 'paroxetine mesylate':ti,ab OR 'paroxia (drug)':ti,ab OR 'paxan':ti,ab OR 'paxil':ti,ab OR 'paxil cr':ti,ab OR 'paxtine':ti,ab OR 'paxxet':ti,ab OR 'pexeva':ti,ab OR 'sereupin':ti,ab OR 'seroxat':ti,ab OR 'setine':ti,ab OR 'si 211103':ti,ab OR 'si211103':ti,ab OR 'solben (drug)':ti,ab OR 'syntopar':ti,ab OR 'tagonis':ti,ab) AND [randomized controlled trial]/lim AND ([article]/lim OR [article in press]/lim) AND [humans]/lim AND [english]/lim AND [embase]/lim——620

#14 ('escitalopram'/exp OR 'escitalopram') AND [randomized controlled trial]/lim AND ([article]/lim OR [article in press]/lim) AND [humans]/lim AND [english]/lim AND [embase]/lim——747

#15 (cipralex:ab,ti OR 'enlift (drug)':ab,ti OR entact:ab,ti OR esciprex:ab,ti OR 'esciprex distab':ab,ti OR 'escitalopram oxalate':ab,ti OR lexapro:ab,ti OR 'lu 26054 0':ab,ti OR 'lu 260540':ab,ti OR lu260540:ab,ti OR premalex:ab,ti OR prilect:ab,ti OR seroplex:ab,ti OR sipralexa:ab,ti OR zecidec:ab,ti OR zocital:ab,ti) AND [randomized controlled trial]/lim AND ([article]/lim OR [article in press]/lim) AND [humans]/lim AND [english]/lim AND [embase]/lim——15

#16 ('fluoxetine'/exp OR 'fluoxetine') AND [randomized controlled trial]/lim AND ([article]/lim OR [article in press]/lim) AND [humans]/lim AND [english]/lim AND [embase]/lim——1579

#17 ('3 (4 trifluoromethylphenoxy) n methyl 3 phenylpropylamine':ab,ti OR '3 n methyl 3 phenyl 3 (4 trifluoromethylphenoxy) propylamine':ab,ti OR actan:ab,ti OR adofen:ab,ti OR afeksin:ab,ti OR 'alzac 20':ab,ti OR andep:ab,ti OR andepin:ab,ti OR ansilan:ab,ti OR 'atd 20':ab,ti OR auroken:ab,ti OR auscap:ab,ti OR bioxetin:ab,ti OR captaton:ab,ti OR 'compound 110140':ab,ti OR daforin:ab,ti OR dagrilan:ab,ti OR depren:ab,ti OR 'deprex (fluoxetine)':ab,ti OR 'deprex leciva':ab,ti OR deprexetin:ab,ti OR deprexin:ab,ti OR deprizac:ab,ti OR deproxin:ab,ti OR diesan:ab,ti OR digassim:ab,ti OR elizac:ab,ti OR exostrept:ab,ti OR felicium:ab,ti OR fldiss:ab,ti OR flotinal:ab,ti OR floxet:ab,ti OR fluctin:ab,ti OR fluctine:ab,ti OR fludac:ab,ti OR flufran:ab,ti OR fluketin:ab,ti OR flunil:ab,ti OR flunirin:ab,ti OR fluohexal:ab,ti OR fluoksetin:ab,ti OR fluoksetyna:ab,ti OR fluox:ab,ti OR 'fluox puren':ab,ti OR fluoxac:ab,ti OR fluoxeren:ab,ti OR fluoxetin:ab,ti OR fluoxetina:ab,ti OR 'fluoxetine hydrochloride':ab,ti OR fluoxifar:ab,ti OR fluoxil:ab,ti OR fluoxone:ab,ti OR 'fluoxone divule':ab,ti OR fluoxtab:ab,ti OR fluronin:ab,ti OR flusac:ab,ti OR flustad:ab,ti OR flutin:ab,ti OR flutine:ab,ti OR 'flux (drug)':ab,ti OR fluxemed:ab,ti OR fluxen:ab,ti OR fluxet:ab,ti OR fluxetil:ab,ti OR fluxetin:ab,ti OR fluxil:ab,ti OR fluxomed:ab,ti OR fluzac:ab,ti OR fokeston:ab,ti OR fontex:ab,ti OR foxetin:ab,ti OR foxtin:ab,ti OR fropine:ab,ti OR fuloren:ab,ti OR gerozac:ab,ti OR ladose:ab,ti OR lanclic:ab,ti OR 'lilly 110140':ab,ti OR 'lilly110140':ab,ti OR lorien:ab,ti OR lovan:ab,ti OR luramon:ab,ti OR 'ly 110140':ab,ti OR ly110140:ab,ti OR magrilan:ab,ti OR margrilan:ab,ti OR meropan:ab,ti OR modipran:ab,ti OR mutan:ab,ti OR 'n methyl 3 phenyl 3 (4 trifluoromethylphenoxy) propylamine':ab,ti OR 'n methyl 3 phenyl 3 [ (alpha, alpha, alpha trifluoro para tolyl) oxy] propylamine':ab,ti OR nopres:ab,ti OR nuzak:ab,ti OR olena:ab,ti OR oxactin:ab,ti OR oxedep:ab,ti OR 'phenylpropylamine, n methyl 3 (4 trifluoromethylphenoxy)':ab,ti OR plazeron:ab,ti OR plinzene:ab,ti OR 'portal (drug)':ab,ti OR pragmaten:ab,ti OR prizma:ab,ti OR proctin:ab,ti OR prodep:ab,ti OR prosac:ab,ti OR prozac:ab,ti OR 'prozac 20':ab,ti OR 'prozac dispersible':ab,ti OR 'prozac weekly':ab,ti OR prozamel:ab,ti OR prozamin:ab,ti OR prozep:ab,ti OR prozit:ab,ti OR psipax:ab,ti OR qualisac:ab,ti OR rapiflux:ab,ti OR reconcile:ab,ti OR reneuron:ab,ti OR rowexetina:ab,ti OR salipax:ab,ti OR sanzur:ab,ti OR sarafem:ab,ti OR sartuzin:ab,ti OR selfemra:ab,ti OR seromex:ab,ti OR seronil:ab,ti OR sinzac:ab,ti OR sofelin:ab,ti OR 'stephadilat s':ab,ti OR xeredien:ab,ti OR zactin:ab,ti OR zepax:ab,ti OR zinovat:ab,ti) AND [randomized controlled trial]/lim AND ([article]/lim OR [article in press]/lim) AND [humans]/lim AND [english]/lim AND [embase]/lim——51

#18 ('sertraline'/exp OR 'sertraline') AND [randomized controlled trial]/lim AND ([article]/lim OR [article in press]/lim) AND [humans]/lim AND [english]/lim AND [embase]/lim——1265

#19 ('1 methylamino 4 (3, 4 dichlorophenyl) tetralin':ab,ti OR '4 (3, 4 dichlorophenyl) 1, 2, 3, 4 tetrahydro n methyl 1 naphthalenamine':ab,ti OR '4 (3, 4 dichlorophenyl) 1, 2, 3, 4 tetrahydro n methylnaphthalen 1 amine':ab,ti OR adjuvin:ab,ti OR altruline:ab,ti OR aremis:ab,ti OR atruline:ab,ti OR besitran:ab,ti OR 'cp 51974':ab,ti OR 'cp 51974 01':ab,ti OR 'cp 51974 1':ab,ti OR 'cp 519741':ab,ti OR cp51974:ab,ti OR 'cp51974 01':ab,ti OR 'cp51974 1':ab,ti OR cp5197401:ab,ti OR cp519741:ab,ti OR dominum:ab,ti OR doxime:ab,ti OR fatral:ab,ti OR fridep:ab,ti OR gladem:ab,ti OR lesefer:ab,ti OR lustral:ab,ti OR 'n methyl 4 (3, 4 dichlorophenyl) 1, 2, 3, 4 tetrahydro 1 naphthylamine':ab,ti OR nudep:ab,ti OR seltra:ab,ti OR serad:ab,ti OR sercerin:ab,ti OR serlain:ab,ti OR serlift:ab,ti OR sertralin:ab,ti OR 'sertraline hydrochloride':ab,ti OR sertranex:ab,ti OR sertranquil:ab,ti OR sosser:ab,ti OR tatig:ab,ti OR tresleen:ab,ti OR zolof:ab,ti OR zoloft:ab,ti OR zosert:ab,ti) AND [randomized controlled trial]/lim AND ([article]/lim OR [article in press]/lim) AND [humans]/lim AND [english]/lim AND [embase]/lim——47

#20 ('fluvoxamine'/exp OR 'fluvoxamine') AND [randomized controlled trial]/lim AND ([article]/lim OR [article in press]/lim) AND [humans]/lim AND [english]/lim AND [embase]/lim——429

#21('5 methoxy 1 [4 (trifluoromethyl) phenyl] 1 pentanone o (2 aminoethyl) oxime':ab,ti OR '5 methoxy 4 (trifluoromethyl) valerophenone o (2 aminoethyl) oxime':ab,ti OR 'du 23000':ab,ti OR du23000:ab,ti OR fluoxamine:ab,ti OR fluroxamine:ab,ti) AND [randomized controlled trial]/lim AND ([article]/lim OR [article in press]/lim) AND [humans]/lim AND [english]/lim AND [embase]/lim——1

#22 #8 OR #9 OR #10 OR #11 OR #12 OR #13 OR #14 OR #15 OR #16 OR #17 OR #18 OR #19 OR #20 OR #21——9643

#23 #7 AND #22——163

Search strategy in Cochrane Central Register of Controlled Trials

#1 stroke[Mesh]——9586

#2 (Strokes OR "Cerebrovascular Accident" OR "Cerebrovascular Accidents" OR "CVA(Cerebrovascular Accident)" OR CVAs OR " Cerebrovascular Apoplexy" OR "Apoplexy, Cerebrovascular" OR "Vascular Accident, Brain" OR "Brain Vascular Accident" OR "Brain Vascular Accidents" OR "Vascular Accidents, Brain" OR "Cerebrovascular Stroke" OR "Cerebrovascular Strokes" OR "Stroke, Cerebrovascular" OR "Strokes, Cerebrovascular" OR Apoplexy OR "Cerebral Stroke" OR "Cerebral Strokes" OR "Stroke, Cerebral" OR " Strokes, Cerebral" OR "Stroke, Acute" OR "Acute Stroke" OR "Acute Strokes" OR "Strokes, Acute" OR "Cerebrovascular Accident, Acute" OR "Acute Cerebrovascular Accident" OR "Acute Cerebrovascular Accidents" OR "Cerebrovascular Accidents, Acute"):ti,ab,kw——58021

#3 "cerebral infarction" [Mesh]——1141

#4 ("Cerebral Infarctions" OR "Infarctions, Cerebral" OR "Infarction, Cerebral" OR "Cerebral Infarct" OR "Cerebral Infarcts" OR "Infarct, Cerebral" OR "Infarcts, Cerebral" OR "Cerebral Infarction, Left Hemisphere" OR "Left Hemisphere, Infarction, Cerebral" OR "Infarction, Left Hemisphere, Cerebral" OR "Left Hemisphere, Cerebral Infarction" OR "Cerebral, Left Hemisphere, Infarction" OR "Infarction, Cerebral, Left Hemisphere" OR "Subcortical Infarction" OR "Infarction, Subcortical" OR "Infarctions, Subcortical" OR "Subcortical Infarctions" OR "Posterior Choroidal Artery Infarction" OR "Anterior Choroidal Artery Infarction" OR "Cerebral Infarction, Right Hemisphere" OR "Right Hemisphere, Cerebral Infarction" OR "Infarction, Right Hemisphere, Cerebral" OR "Infarction, Cerebral, Right Hemisphere" OR "Right Hemisphere, Infarction, Cerebral" OR "Cerebral, Right Hemisphere, Infarction"):ti,ab,kw——428

#5 "brain infarction" [Mesh]——1270

#6 ("Brain Infarctions" OR "Infarction, Brain" OR "Infarctions, Brain" OR "Brain Infarct" OR "Brain Infarcts" OR "Infarct, Brain" OR "Infarcts, Brain" OR "Anterior Circulation Brain Infarction" OR "Infarction, Brain, Anterior Circulation" OR "Infarction, Anterior Circulation, Brain" OR "Anterior Circulation Infarction, Brain" OR "Brain Infarction, Anterior Circulation" OR "Venous Infarction, Brain" OR "Brain Venous Infarction" OR "Brain Venous Infarctions" OR "Infarction, Brain Venous" OR "Infarctions, Brain Venous" OR " Venous Infarctions, Brain" OR "Brain Infarction, Venous" OR "Brain Infarctions, Venous" OR "Infarction, Venous Brain" OR "Infarctions, Venous Brain" OR "Venous Brain Infarction" OR "Venous Brain Infarctions" OR "Anterior Cerebral Circulation Infarction" OR "Infarction, Anterior Cerebral Circulation" OR "Brain Infarction, Posterior Circulation" OR "Posterior Circulation Infarction, Brain" OR "Posterior Circulation Brain Infarction" OR "Infarction, Brain, Posterior Circulation" OR "Infarction, Posterior Circulation, Brain"):ti,ab,kw——349

#7 "cerebral hemorrhage" [Mesh]——956

#8 ("Hemorrhage, Cerebrum" OR "Cerebrum Hemorrhage" OR "Cerebrum Hemorrhages" OR "Hemorrhages, Cerebrum" OR "Cerebral Parenchymal Hemorrhage" OR "Cerebral Parenchymal Hemorrhages" OR "Hemorrhage, Cerebral Parenchymal " OR "Hemorrhages, Cerebral Parenchymal" OR "Parenchymal Hemorrhage, Cerebral" OR "Parenchymal Hemorrhages, Cerebral" OR "Intracerebral Hemorrhage" OR "Hemorrhage, Intracerebral" OR "Hemorrhages, Intracerebral" OR "Intracerebral Hemorrhages" OR "Hemorrhage, Cerebral" OR "Cerebral Hemorrhages" OR "Hemorrhages, Cerebral" OR "Brain Hemorrhage, Cerebral" OR "Brain Hemorrhages, Cerebral" OR "Cerebral Brain Hemorrhage" OR "Cerebral Brain Hemorrhages" OR "Hemorrhage, Cerebral Brain" OR "Hemorrhages, Cerebral Brain"):ti,ab,kw——2233

#9 "Intracranial Hemorrhages" [Mesh]——1898

#10 ("Hemorrhages, Intracranial" OR "Intracranial Hemorrhage" OR "Hemorrhage, Intracranial" OR "Posterior Fossa Hemorrhage" OR "Hemorrhage, Posterior Fossa" OR "Hemorrhages, Posterior Fossa" OR "Posterior Fossa Hemorrhages" OR "Brain Hemorrhage" OR "Brain Hemorrhages" OR "Hemorrhage, Brain" OR "Hemorrhages, Brain"):ti,ab,kw——4620

#11 #1 OR #2 OR #3 OR #4 OR #5 OR #6 OR #7 OR #8 OR #9 OR #10——62256

#12 "Serotonin Uptake Inhibitors"[Mesh]——2694

#13 ("5-Hydroxytryptamine Uptake Inhibitors" OR "5 Hydroxytryptamine Uptake Inhibitors" OR "Inhibitors, 5-HT Uptake" OR "Inhibitors, 5 HT Uptake" OR "Inhibitors, 5-Hydroxytryptamine Uptake" OR "Inhibitors, 5 Hydroxytryptamine Uptake" OR "Inhibitors, Serotonin Reuptake" OR "Reuptake Inhibitors, Serotonin" OR "Serotonin Reuptake Inhibitors" OR "Uptake Inhibitors, 5-HT" OR "Uptake Inhibitors, 5 HT" OR "Uptake Inhibitors, 5-Hydroxytryptamine" OR "Uptake Inhibitors, 5 Hydroxytryptamine" OR "Uptake Inhibitors, Serotonin" OR "5-HT Uptake Inhibitors" OR "5 HT Uptake Inhibitors" OR "Inhibitors, Serotonin Uptake" OR "Selective Serotonin Reuptake Inhibitors"):ti,ab,kw——1467

#14 Citalopram[Mesh]——1451

#15 (Cytalopram OR Seropram OR Escitalopram OR Celexa OR "Lu-10-171" OR Lu10171 OR "Citalopram Hydrobromide" OR "Escitalopram Oxalate" OR Lexapro) :ti,ab,kw——1733

#16 Fluoxetine[Mesh]——1424

#17 (Fluoxetin OR "N-Methyl-gamma-(4-(trifluoromethyl)phenoxy)benzenepropanamine" OR "Lilly-110140" OR "Lilly 110140" OR Lilly110140 OR Sarafem OR "Fluoxetine Hydrochloride" OR Prozac):ti,ab,kw——176

#18 Paroxetine[Mesh]——972

#19 (Aropax OR "BRL-29060" OR "BRL 29060" OR BRL29060 OR "FG-7051" OR "FG 7051" OR FG7051 OR "Paroxetine Acetate" OR Seroxat OR "Paroxetine Hydrochloride Anhydrous" OR "Paroxetine Maleate" OR "Paroxetine, cis-(+)-Isomer" OR "Paroxetine, cis-(-)-Isomer" OR "Paroxetine, trans-(+)-Isomer" OR Paxil OR "Paroxetine Hydrochloride Hemihydrate" OR "Paroxetine Hydrochloride, Hemihydrate" OR "Paroxetine Hydrochloride"):ti,ab,kw——124

#20 Sertraline[Mesh]——1007

#21 (Zoloft OR Altruline OR Lustral OR "Apo-Sertraline" OR "Apo Sertraline" OR Aremis OR Besitran OR Sealdin OR Gladem OR "Novo-Sertraline" OR "Novo Sertraline" OR "ratio-Sertraline" OR "ratio Sertraline" OR "Rhoxal-sertraline" OR "Rhoxal sertraline" OR "Sertraline Hydrochloride" OR "Hydrochloride, Sertraline" OR "Sertraline Hydrochloride (1S-cis)-Isomer" OR "Gen-Sertraline" OR "Gen Sertraline"):ti,ab,kw——169

#22 Fluvoxamine[Mesh]——389

#23 (Fluvoxadura OR "Fluvoxamin AL" OR "Fluvoxamin beta" OR "Fluvoxamin Stada" OR "Fluvoxamin-neuraxpharm" OR "Fluvoxamin neuraxpharm" OR "Fluvoxamin-ratiopharm" OR "Fluvoxamin ratiopharm" OR "ratio-Fluvoxamine" OR "ratio Fluvoxamine" OR "Fluvoxamina Geminis" OR "Geminis, Fluvoxamina" OR "Fluvoxamine Maleate" OR "Fluvoxamine Maleate, (E)-Isomer" OR "Fluvoxamine, (Z)-Isomer" OR Luvox OR Floxyfral OR Fevarin OR Dumirox OR Faverin OR "Novo-Fluvoxamine" OR "Novo Fluvoxamine" OR "Nu-Fluvoxamine" OR "Nu Fluvoxamine" OR "PMS-Fluvoxamine" OR "PMS Fluvoxamine" OR Desiflu OR "DU-23000" OR "DU 23000" OR DU23000):ti,ab,kw——62

#24 #12 OR #13 OR #14 OR #15 OR #16 OR #17 OR #18 OR #19 OR #20 OR #21 OR #22 OR #23——7596

#25 #11 AND #24——195

Search strategy in Scopus

#1 TITLE-ABS(stroke OR Strokes OR "Cerebrovascular Accident" OR "Cerebrovascular Accidents" OR "CVA(Cerebrovascular Accident)" OR CVAs OR " Cerebrovascular Apoplexy" OR "Apoplexy, Cerebrovascular" OR "Vascular Accident, Brain" OR "Brain Vascular Accident" OR "Brain Vascular Accidents" OR "Vascular Accidents, Brain" OR "Cerebrovascular Stroke" OR "Cerebrovascular Strokes" OR "Stroke, Cerebrovascular" OR "Strokes, Cerebrovascular" OR Apoplexy OR "Cerebral Stroke" OR "Cerebral Strokes" OR "Stroke, Cerebral" OR " Strokes, Cerebral" OR "Stroke, Acute" OR "Acute Stroke" OR "Acute Strokes" OR "Strokes, Acute" OR "Cerebrovascular Accident, Acute" OR "Acute Cerebrovascular Accident" OR "Acute Cerebrovascular Accidents" OR "Cerebrovascular Accidents, Acute")——350498

#2 TITLE-ABS("cerebral infarction" OR "Cerebral Infarctions" OR "Infarctions, Cerebral" OR "Infarction, Cerebral" OR "Cerebral Infarct" OR "Cerebral Infarcts" OR "Infarct, Cerebral" OR "Infarcts, Cerebral" OR "Cerebral Infarction, Left Hemisphere" OR "Left Hemisphere, Infarction, Cerebral" OR "Infarction, Left Hemisphere, Cerebral" OR "Left Hemisphere, Cerebral Infarction" OR "Cerebral, Left Hemisphere, Infarction" OR "Infarction, Cerebral, Left Hemisphere" OR "Subcortical Infarction" OR "Infarction, Subcortical" OR "Infarctions, Subcortical" OR "Subcortical Infarctions" OR "Posterior Choroidal Artery Infarction" OR "Anterior Choroidal Artery Infarction" OR "Cerebral Infarction, Right Hemisphere" OR "Right Hemisphere, Cerebral Infarction" OR "Infarction, Right Hemisphere, Cerebral" OR "Infarction, Cerebral, Right Hemisphere" OR "Right Hemisphere, Infarction, Cerebral" OR "Cerebral, Right Hemisphere, Infarction")——22597

#3 TITLE-ABS("brain infarction" OR "Brain Infarctions" OR "Infarction, Brain" OR "Infarctions, Brain" OR "Brain Infarct" OR "Brain Infarcts" OR "Infarct, Brain" OR "Infarcts, Brain" OR "Anterior Circulation Brain Infarction" OR "Infarction, Brain, Anterior Circulation" OR "Infarction, Anterior Circulation, Brain" OR "Anterior Circulation Infarction, Brain" OR "Brain Infarction, Anterior Circulation" OR "Venous Infarction, Brain" OR "Brain Venous Infarction" OR "Brain Venous Infarctions" OR "Infarction, Brain Venous" OR "Infarctions, Brain Venous" OR " Venous Infarctions, Brain" OR "Brain Infarction, Venous" OR "Brain Infarctions, Venous" OR "Infarction, Venous Brain" OR "Infarctions, Venous Brain" OR "Venous Brain Infarction" OR "Venous Brain Infarctions" OR "Anterior Cerebral Circulation Infarction" OR "Infarction, Anterior Cerebral Circulation" OR "Brain Infarction, Posterior Circulation" OR "Posterior Circulation Infarction, Brain" OR "Posterior Circulation Brain Infarction" OR "Infarction, Brain, Posterior Circulation" OR "Infarction, Posterior Circulation, Brain")——4857

#4 TITLE-ABS("cerebral hemorrhage" OR "Hemorrhage, Cerebrum" OR "Cerebrum Hemorrhage" OR "Cerebrum Hemorrhages" OR "Hemorrhages, Cerebrum" OR "Cerebral Parenchymal Hemorrhage" OR "Cerebral Parenchymal Hemorrhages" OR "Hemorrhage, Cerebral Parenchymal " OR "Hemorrhages, Cerebral Parenchymal" OR "Parenchymal Hemorrhage, Cerebral" OR "Parenchymal Hemorrhages, Cerebral" OR "Intracerebral Hemorrhage" OR "Hemorrhage, Intracerebral" OR "Hemorrhages, Intracerebral" OR "Intracerebral Hemorrhages" OR "Hemorrhage, Cerebral" OR "Cerebral Hemorrhages" OR "Hemorrhages, Cerebral" OR "Brain Hemorrhage, Cerebral" OR "Brain Hemorrhages, Cerebral" OR "Cerebral Brain Hemorrhage" OR "Cerebral Brain Hemorrhages" OR "Hemorrhage, Cerebral Brain" OR "Hemorrhages, Cerebral Brain")——23150

#5 TITLE-ABS("Intracranial Hemorrhages" OR "Hemorrhages, Intracranial" OR "Intracranial Hemorrhage" OR "Hemorrhage, Intracranial" OR "Posterior Fossa Hemorrhage" OR "Hemorrhage, Posterior Fossa" OR "Hemorrhages, Posterior Fossa" OR "Posterior Fossa Hemorrhages" OR "Brain Hemorrhage" OR "Brain Hemorrhages" OR "Hemorrhage, Brain" OR "Hemorrhages, Brain")——15535

#6 #1 OR #2 OR #3 OR #4 OR #5——390368

#7 TITLE-ABS("Serotonin Uptake Inhibitors" OR "5-Hydroxytryptamine Uptake Inhibitors" OR "5 Hydroxytryptamine Uptake Inhibitors" OR "Inhibitors, 5-HT Uptake" OR "Inhibitors, 5 HT Uptake" OR "Inhibitors, 5-Hydroxytryptamine Uptake" OR "Inhibitors, 5 Hydroxytryptamine Uptake" OR "Inhibitors, Serotonin Reuptake" OR "Reuptake Inhibitors, Serotonin" OR "Serotonin Reuptake Inhibitors" OR "Uptake Inhibitors, 5-HT" OR "Uptake Inhibitors, 5 HT" OR "Uptake Inhibitors, 5-Hydroxytryptamine" OR "Uptake Inhibitors, 5 Hydroxytryptamine" OR "Uptake Inhibitors, Serotonin" OR "5-HT Uptake Inhibitors" OR "5 HT Uptake Inhibitors" OR "Inhibitors, Serotonin Uptake" OR "Selective Serotonin Reuptake Inhibitors")——16739

#8 TITLE-ABS(Citalopram OR Cytalopram OR Seropram OR Escitalopram OR Celexa OR "Lu-10-171" OR Lu10171 OR "Citalopram Hydrobromide" OR "Escitalopram Oxalate" OR Lexapro OR Fluoxetine OR Fluoxetin OR "N-Methyl-gamma-(4-(trifluoromethyl)phenoxy)benzenepropanamine" OR "Lilly-110140" OR "Lilly 110140" OR Lilly110140 OR Sarafem OR "Fluoxetine Hydrochloride" OR Prozac OR Paroxetine OR Aropax OR "BRL-29060" OR "BRL 29060" OR BRL29060 OR "FG-7051" OR "FG 7051" OR FG7051 OR "Paroxetine Acetate" OR Seroxat OR "Paroxetine Hydrochloride Anhydrous" OR "Paroxetine Maleate" OR "Paroxetine, cis-(+)-Isomer" OR "Paroxetine, cis-(-)-Isomer" OR "Paroxetine, trans-(+)-Isomer" OR Paxil OR "Paroxetine Hydrochloride Hemihydrate" OR "Paroxetine Hydrochloride, Hemihydrate" OR "Paroxetine Hydrochloride" OR Sertraline OR Zoloft OR Altruline OR Lustral OR "Apo-Sertraline" OR "Apo Sertraline" OR Aremis OR Besitran OR Sealdin OR Gladem OR "Novo-Sertraline" OR "Novo Sertraline" OR "ratio-Sertraline" OR "ratio Sertraline" OR "Rhoxal-sertraline" OR "Rhoxal sertraline" OR "Sertraline Hydrochloride" OR "Hydrochloride, Sertraline" OR "Sertraline Hydrochloride (1S-cis)-Isomer" OR "Gen-Sertraline" OR "Gen Sertraline" OR Fluvoxamine OR Fluvoxadura OR "Fluvoxamin AL" OR "Fluvoxamin beta" OR "Fluvoxamin Stada" OR "Fluvoxamin-neuraxpharm" OR "Fluvoxamin neuraxpharm" OR "Fluvoxamin-ratiopharm" OR "Fluvoxamin ratiopharm" OR "ratio-Fluvoxamine" OR "ratio Fluvoxamine" OR "Fluvoxamina Geminis" OR "Geminis, Fluvoxamina" OR "Fluvoxamine Maleate" OR "Fluvoxamine Maleate, (E)-Isomer" OR "Fluvoxamine, (Z)-Isomer" OR Luvox OR Floxyfral OR Fevarin OR Dumirox OR Faverin OR "Novo-Fluvoxamine" OR "Novo Fluvoxamine" OR "Nu-Fluvoxamine" OR "Nu Fluvoxamine" OR "PMS-Fluvoxamine" OR "PMS Fluvoxamine" OR Desiflu OR "DU-23000" OR "DU 23000" OR DU23000)——31334

#9 #7 OR #8——40382

#10 TITLE-ABS("randomized controlled trial" OR "controlled clinical trial" OR "controlled trial, randomized" OR "randomised controlled trial" OR "randomized controlled study" OR "controlled study" OR "multicenter study")——324847

#11 #6 AND #9 AND #10 AND  (LIMIT-TO(DOCTYPE , "ar")) AND (LIMIT-TO(LANGUAGE , "English"))——57

Search strategy in ScienceDirect

(stroke OR "cerebrovascular accident" OR "cerebral infarction" OR "brain infarction" OR "cerebral hemorrhage" OR "intracranial hemorrhages") AND ("serotonin uptake inhibitors" OR citalopram OR escitalopram OR fluoxetine OR paroxetine OR sertraline OR fluvoxamine)——295

Search strategy in ClinicalTrials.gov

(stroke OR "cerebrovascular accident" OR "cerebral infarction" OR "brain infarction" OR "cerebral hemorrhage" OR "intracranial hemorrhages") AND ("serotonin uptake inhibitors" OR citalopram OR escitalopram OR fluoxetine OR paroxetine OR sertraline OR fluvoxamine) AND (interventional studies(clinical trials)) AND (adult and older adult (18 yrs & older)) AND (completed studies)——12
