# Supplementary material for: The Efficacy and Tolerability of Selective Serotonin Reuptake Inhibitors for Motor Recovery in Non-depressed Patients After Acute Stroke: A Meta-Analysis
Source: Front Neurol. 2021 Oct 20;12:749322. doi: 10.3389/fneur.2021.749322 (PMC8564176; doi:10.3389/fneur.2021.749322)
Supplement: Supplementary file 2 [file Table_2.DOCX]

**Supplementary S2 file.** Sensitivity analyses


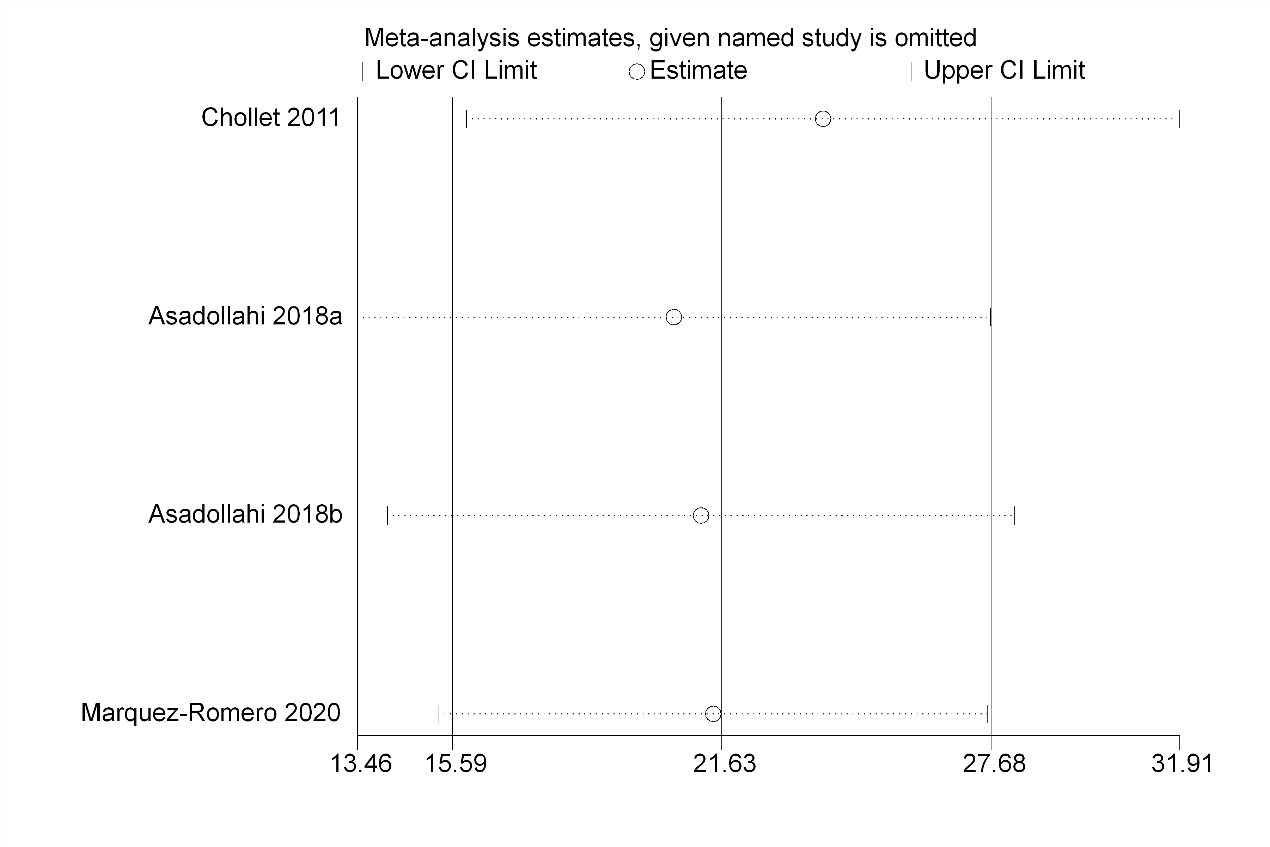


FMMS-endpoint score


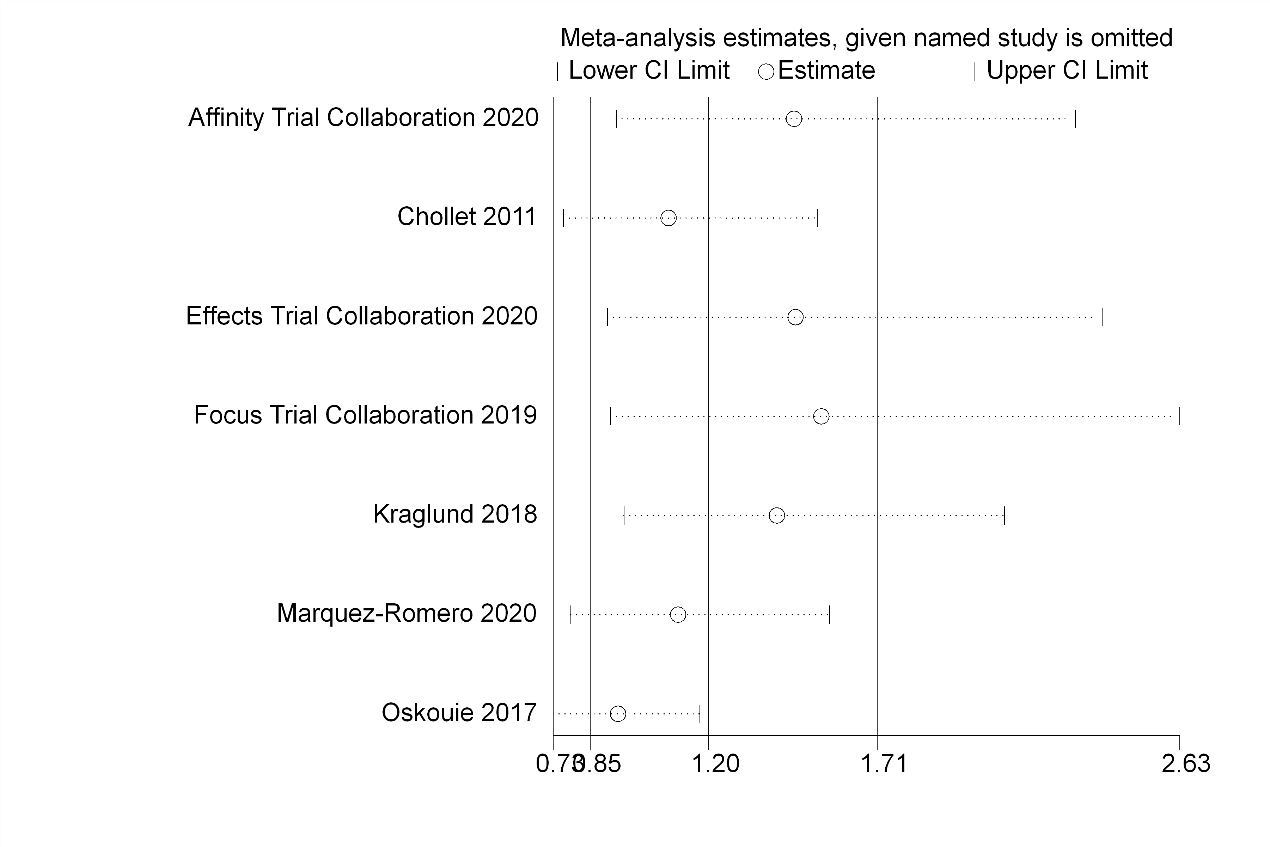
The proportion of the number of people with mRS score 0-2


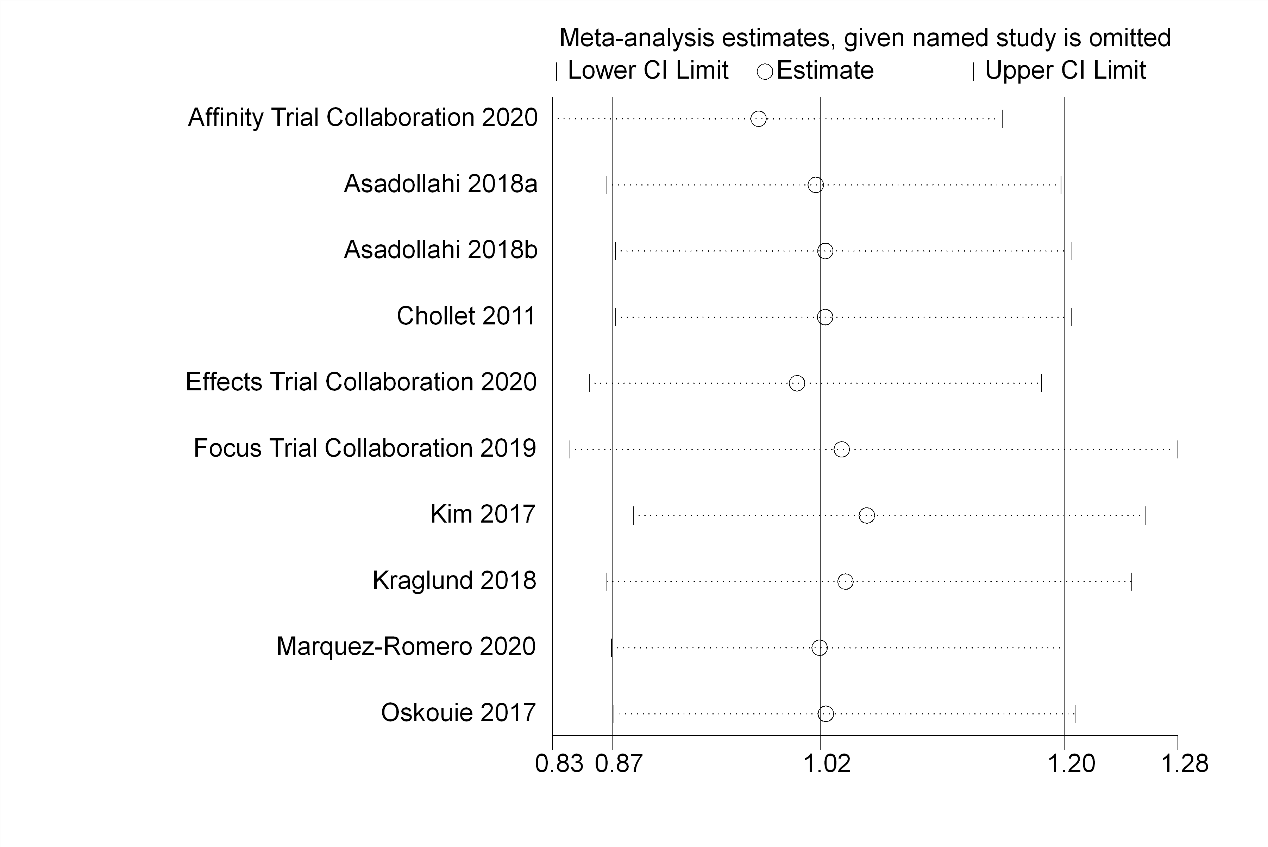


total withdrawal rate
